# Bright monomeric near-infrared fluorescent proteins as tags and biosensors for multiscale imaging

**DOI:** 10.1038/ncomms12405

**Published:** 2016-08-19

**Authors:** Daria M. Shcherbakova, Mikhail Baloban, Alexander V. Emelyanov, Michael Brenowitz, Peng Guo, Vladislav V. Verkhusha

**Affiliations:** 1 Department of Anatomy and Structural Biology, Albert Einstein College of Medicine, Bronx, New York 10461, USA; 2 Gruss-Lipper Biophotonics Center, Albert Einstein College of Medicine, Bronx, New York 10461, USA; 3 Department of Cell Biology, Albert Einstein College of Medicine, Bronx, New York 10461, USA; 4 Department of Biochemistry, Albert Einstein College of Medicine, Bronx, New York 10461, USA; 5 Analytical Imaging Facility, Albert Einstein College of Medicine, Bronx, New York 10461, USA

## Abstract

Monomeric near-infrared (NIR) fluorescent proteins (FPs) are in high demand as protein tags and components of biosensors for deep-tissue imaging and multicolour microscopy. We report three bright and spectrally distinct monomeric NIR FPs, termed miRFPs, engineered from bacterial phytochrome, which can be used as easily as GFP-like FPs. miRFPs are 2–5-fold brighter in mammalian cells than other monomeric NIR FPs and perform well in protein fusions, allowing multicolour structured illumination microscopy. miRFPs enable development of several types of NIR biosensors, such as for protein–protein interactions, RNA detection, signalling cascades and cell fate. We demonstrate this by engineering the monomeric fluorescence complementation reporters, the IκBα reporter for NF-κB pathway and the cell cycle biosensor for detection of proliferation status of cells in culture and in animals. miRFPs allow non-invasive visualization and detection of biological processes at different scales, from super-resolution microscopy to *in vivo* imaging, using the same probes.

## Abstract (web summary)

Near-infrared fluorescent proteins are non-invasive probes for deep tissue imaging, but because of the dimeric state they perform poorly in protein labelling. Here, the authors engineered three spectrally resolvable monomeric near-infrared probes with improved brightness for multiscale imaging.

## Graphical abstract

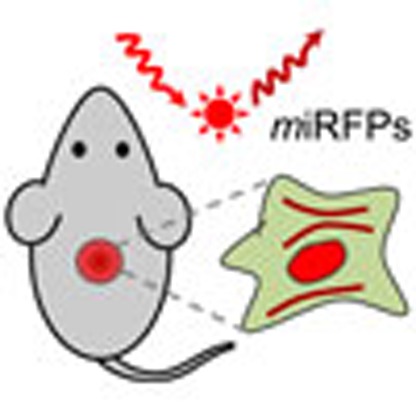

Non-invasive *in vivo* imaging requires near-infrared (NIR) fluorescent probes. Recent development of genetically encoded fluorescent proteins (FPs) from bacterial phytochrome photoreceptors (BphP) has significantly advanced deep-tissue and whole-body imaging^1^. In contrast to far-red green fluorescent protein (GFP)-like FPs, BphP-based FPs are excited and fluoresce close to or within an NIR tissue transparency ‘optical window' (approximately 650–900 nm) where background autofluorescence is low, light scattering is reduced, and combined absorption of haemoglobin, melanin and water is minimal^2^.

NIR fluorescence of BphP-based FPs results from an incorporation of the most red-shifted natural chromophore, biliverdin IXa (BV)^1^,^3^,^4^, that is similar to their parental BphPs^5^,^6^. Fortunately, BV is abundant in eukaryotes, including mammals, as an intermediate of haem degradation pathway to bilirubin^7^,^8^. In wild-type BphPs, light absorption results in BV isomerization and conformational changes of the protein backbone, leading to activation of an output effector domain. In engineered NIR FPs, the photoisomerization is blocked and the other non-radiative energy dissipation pathways are suppressed by truncation of BphPs to the chromophore-binding PAS-GAF domains and by introducing of amino-acid substitutions in the chromophore immediate environment^1^,^9^.

Although BphP-based NIR FPs are now widely used in many areas of basic and translational research, including cancer studies, stem cell biology, neuroscience and parasitology, these FPs are mainly serve as passive whole-cell labels for non-invasive *in vivo* imaging^5^. So far these NIR FPs had the limited use in monitoring of active cellular processes in animals, such as activation of signalling cascades and protein–protein interactions (PPIs). A development of active NIR reporters and biosensors, which respond to cellular events and consequently change their fluorescence, has been hampered by a lack of bright monomeric NIR FPs as building blocks for these sensors. The monomeric NIR FPs are also required to label (tag) intracellular proteins. Currently available monomeric far-red GFP-like FPs, including mKate2 (ref. 10), TagRFP657 (ref. 11), mCardinal and mNeptune2.5 (ref. 12), are suboptimal for deep-tissue imaging because their excitation maxima do not exceed 611 nm.

Current BphP-based NIR FPs have limitations and cannot be used to label proteins and to build NIR biosensors. There are three characteristics of NIR FPs, which are crucial to consider for their applications^1^. The first one is an effective brightness of NIR FP in mammalian cells, which depends on its molecular brightness, intracellular stability, efficiency of BV incorporation and cell expression level. In contrast to GFP-like FPs, the effective brightness of BphP-based NIR FPs does not always correlate with their molecular brightness^1^. Decreased cellular fluorescence of some NIR FPs results from a low specificity of BV binding and a competition between BV and other haem-derived compounds, including protoporphyrin IX, for binding to NIR FP apoproteins^13^,^14^. The second characteristic to consider is an oligomeric state of FPs. Only monomeric FPs can be used in protein fusions without interference with functionality of the tagged protein partner^15^. The third characteristic is the spectral properties of NIR FPs. Spectrally distinct NIR FPs are required for biosensors and for multicolour NIR labelling.

Among the reported BphP-based FPs, five spectrally distinct NIR FPs, iRFP670, iRFP682, iRFP702, iRFP713 and iRFP720 (refs 1, 4, 16) fully rely on endogenous BV and do not require its external supply or co-expression of haem oxygenase (HO). Therefore, these proteins can be used as easy as GFP-like FPs by delivering a single gene to cells. Importantly, possible endogenous BV concentration variability does not influence performance of iRFPs. Indeed, iRFP713 fluorescence was observed in all tissues of two iRFP713-transgenic mouse lines^8^. In both mouse lines, the iRFP713 fluorescence intensity was generally uniform in almost all organs and tissues, with slightly higher expression levels in liver, lungs and pancreas. However, iRFPs are dimers and can mainly serve for labelling of organelles and whole cells.

The first monomeric BphP-based FP, IFP1.4 (ref. 3), is dim and do not fluoresce without a BV supply. Moreover, it forms dimers, as was found recently^17^. Its brighter version IFP2.0 (ref. 18) was also found to be dimeric^1^,^17^. Previously reported monomeric FPs, Wi-Phy^9^ and IFP1.4rev^19^, were characterized only *in vitro*^9^,^19^. Recently reported monomeric mIFP^17^, which is the only one monomeric FP tested in cellular fusions, is dimmer than dimeric iRFPs and requires a supply of BV via co-expression of BV-producing enzyme, HO. Also, a lack of spectrally distinct versions of monomeric BphP-based FPs prevents two-colour NIR protein labelling and a development of NIR reporters and biosensors.

Here we report a set of three bright spectrally distinct monomeric NIR FPs, called miRFPs, which fully rely on endogenous BV to fluoresce in mammalian cells and mammals. We demonstrate a use of miRFPs in a wide range of NIR protein tags, reporters and biosensors. First, we created a set of miRFP protein fusions and showed that they can be imaged using common diffraction-limited and super-resolution microscopy. Second, using miRFPs as scaffolds, we developed spectrally distinct monomeric bimolecular fluorescence complementation (BiFC) reporters for PPIs and for low-background RNA imaging. Third, we demonstrated a use of miRFPs to develop NIR reporters for signalling cascades and cell fate. Specifically, we designed NIR IkBa and NIR cell cycle reporters and showed that they perform well in applications across scales: from microscopy and flow cytometry to whole-body imaging.

## Results

### Molecular evolution and screening for miRFPs

**Figure 1:**
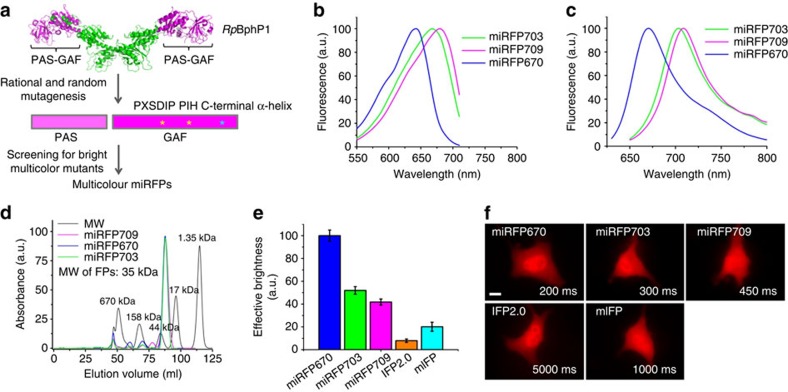
Development and characterization of three monomeric miRFPs. (**a**) Schematics of directed molecular evolution resulted in three monomeric miRFPs. The chromophore-binding PAS-GAF domains, which are not involved in dimerization of *Rp*BphP1, were used as a starting point. To exclude formation of even weak dimers, we mutated residues in the C-terminal α-helix in the GAF domain. To obtain spectrally distinct variants, we mutated residues 201 and 202 in the -PXSDIP- motif and residue 253 in the -PIH- motif in the GAF domain. (**b**) Fluorescence excitation spectra of engineered miRFP670, miRFP703 and miRFP709. (**c**) Fluorescence emission spectra of miRFPs. (**d**) Size exclusion chromatography of miRFPs and indicated molecular weight standards. Apparent molecular weight of all miRFPs was ∼35 kDa. (**e**) Brightness of live HeLa cells transiently transfected with several BphP-based NIR FPs analysed by flow cytometry. The NIR fluorescence intensity was normalized to transfection efficiency (fluorescence of co-transfected EGFP), to excitation efficiency of each FP with 635 nm laser, and to fluorescence signal of each FP in the emission filter. The NIR effective brightness of miRFP670 was assumed to be 100%. Error bars, s.d. (*n*=3; transfection experiments). (**f**) Representative fluorescence images of several BphP-based NIR FPs in live HeLa cells. Acquisition time for each image is indicated. Scale bar, 10 μm.

To engineer miRFPs, we chose the chromophore-binding PAS-GAF domains of *Rp*BphP1 as a starting point for molecular evolution. Structure of the full-length dimeric *Rp*BphP1 (ref. 20) indicates that the PAS-GAF domains do not participate in a dimer interaction (Fig. 1a). We first randomly mutated Asp201 and Ile202 residues, which were shown to stabilize BV chromophore and to increase fluorescence quantum yield^9^,^16^. Then, we subjected the PAS-GAF domains to random mutagenesis with screening in *Escherichia coli* bacteria co-expressing HO for BV production, as described^16^.

Since NIR FP brightness in the HO overexpressing bacteria does not always correlate with the brightness of the proteins in mammalian cells where BV concentration is lower, we tested effective brightness of protein mutants, found during each round of molecular evolution in bacteria, in mammalian cells and discarded the NIR FP variants with the low brightness.

To develop truly monomeric FPs, we specifically focused on residues in the C-terminal α-helix of the GAF domain, which formed dimerization interface in other NIR FPs^9^. To exclude formation of even weak dimers, we additionally mutated residues in the C-terminal α-helix of the mutants obtained after the fourth round. Then, to select the monomeric variants, we tested the bright variants using a size exclusion chromatography and further verified their performance in α-tubulin fusions in mammalian cells.

To develop spectrally distinct FPs, we applied the reported rational design strategy^21^. It has been shown that blue-shifted BphP-based NIR FPs can be obtained by substituting the Ile residue with Cys in the -PIH- motif in the GAF domain^21^. This Cys residue is able to form a covalent bond with BV, resulting in ∼40 nm spectral blue-shift. By replacing this Cys with original Ile, the blue-shifted NIR FP can be converted to standard red-shifted NIR FP with BV bound to a conserved Cys residue in the PAS domain. Earlier we also found that residues in positions 201 and 202 in -P*X*SDIP- motif are not only involved in stabilization of BV chromophore but additionally influence spectral properties of FPs^16^.

As a result, we have obtained miRFP670 (excitation/emission at 642/670 nm) having Cys in the GAF domain and its dimmer version miRFP670v1 (Table 1). By changing Cys back to Ile and mutating residues 201 and 202, we found two red-shifted variants, miRFP703 (excitation/emission at 673/703 nm) and miRFP709 (excitation/emission at 683/709 nm) (Table 1, Supplementary Fig. 1). These three miRFPs exhibit excitation and emission maxima covering the spectral ranges of 642–683 nm and 670–709 nm, respectively (Fig. 1b,c). Absorbance of miRFPs was characterized by small side peaks at 280 nm (protein absorbance) and 390 nm (so called Soret band common to all tetrapyrrole compounds) and by large main peaks at 644, 676 and 685 nm, indicative of the efficient BV incorporation (Supplementary Fig. 2a). Monomeric state of miRFPs was determined by size exclusion chromatography at 1 mg ml^−1^, as well as at concentrations as high as 30 mg ml^−1^ (Fig. 1d, Supplementary Fig. 2b,c). We also confirmed the monomeric state of miRFPs in side-by-side comparison with dimeric IFP2.0 and iRFP713 using size exclusion chromatography and analytical ultracentrifugation (Supplementary Figs 3 and 4).

### Detailed characterization of miRFPs

Molecular brightness (the product of a molar extinction coefficient and a quantum yield) of miRFP670 and miRFP703 was higher than that of dimeric iRFP713, and molecular brightness of miRFP709 was close to that of iRFP713 (Table 1). Interestingly, miRFP670 exhibited a fluorescence quantum yield of 14%, which is the highest for available BphP-based FPs with BV chromophore.

miRFPs were brightest at pH values between 5 and 8, and all had pKa of 4.5 (Supplementary Fig. 2d). All miRFPs exhibited several-fold higher photostability than mIFP in mammalian cells (Supplementary Fig. 2e, Table 1).

We further studied an effective brightness of miRFPs in live mammalian cells. Although less bright than dimeric iRFPs (Table 1), all miRFPs considerably outperformed IFP2.0 and mIFP. The dimmest miRFP709 was more than twofold brighter and the brightest miRFP670 was more than fivefold brighter than mIFP in HeLa cells (Fig. 1e,f). Cell images indicate homogenous distribution of miRFPs and absence of intracellular aggregates. miRFPs require less exposure time than mIFP or IFP2.0 to reach comparative cellular brightness (Fig. 1f). The effective brightness of miRFP703 was also higher than mIFP in other mammalian cells including HEK293, U87, U2OS, Cos-1 (Supplementary Fig. 5). Thus, possible variability of BV levels in different cell lines does not influence the performance of miRFPs.

To study maturation of miRFP proteins, which includes protein synthesis, protein folding and BV chromophore binding, we monitored the NIR fluorescence growth in *E. coli* bacteria on a pulse-chase induction of the protein expression. The half-times of this process were ∼190 min for all three miRFPs (Supplementary Fig. 6a). Next, we separately studied kinetics of BV binding and its dependence on BV concentration. For this, we measured the kinetics of assembly of purified miRFP670 apoprotein with different concentrations of free BV *in vitro* (Supplementary Fig. 6b). The binding half-times for 15 μM apoprotein and 0.1, 1 and 10 μM of BV were 3.5, 1.8, and 1.2 min, respectively. At all BV concentrations, the miRFP670 holoprotein was fully assembled in 20 min. We concluded that miRFPs mature relatively fast, with the protein folding being the slowest step.

To test for cytotoxicity, we monitored stability of miRFP fluorescence over cell generations^22^. This characteristic is crucial for long-term FP applications *in vivo*. Cell labelling typically employs strong promoters, and consequently cells containing high levels of FPs may show growth defects or instability of FP expression^22^,^23^. Preclonal mixtures of HEK293 cells stably expressing miRFPs under CMV promoter were analysed before and after three weeks of culturing. The cells retained >75% of their initial fluorescence intensity (Supplementary Fig. 7) that is comparable to dimeric iRFPs^16^ and to the least cytotoxic far-red GFP-like protein, E2-Crimson^4^,^24^.

We found that miRFPs also can be imaged in primary cell cultures such as neurons (Supplementary Fig. 8). Rat hippocampal neurons transfected with miRFPs exhibited bright homogenous fluorescence without HO co-expression or supplying of exogenous biliverdin, in contrast to IFP2.0 and mIFP, which required the co-expression of HO in neuronal cells^17^,^18^.

### miRFPs in conventional and super-resolution microscopy

**Figure 2:**
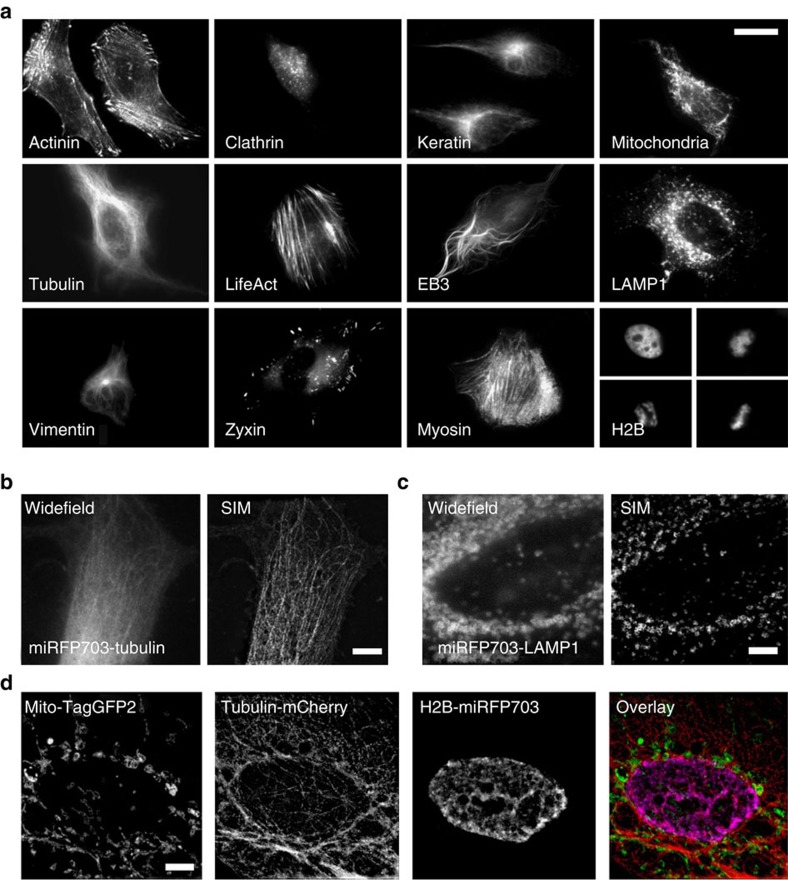
miRFP fusions visualized by widefield and super-resolution microscopy. (**a**) Live HeLa cells transiently transfected with the miRFP703 N- and C-terminal fusion constructs. The N-terminal fusions are α-actinin, keratin, vimentin and tubulin-binding EB3, mitochondrial, focal adhesion protein zyxin, lysosomal membrane glycoprotein LAMP1, vesicular protein clathrin, actin-binding LifeAct and histone H2B. The C-terminal fusions are α-tubulin and myosin. (**b**,**c**) Widefield and structured illumination microscopy (SIM) imaging of fixed HeLa cells expressing α-tubulin (**b**) and LAMP1 (**c**) labelled with miRFP703. (**d**) Three-colour SIM of fixed HeLa cells expressing mitochondria labelled with TagGFP2, α-tubulin labelled with mCherry and H2B labelled with miRFP703. Scale bar, 5 μm.

To test the performance of miRFPs as protein tags, we expressed several miRFP fusions in mammalian cells. Both N- and C-terminal fusions localized properly, including those associated with or forming filaments, such as keratin, α-tubulin, α-actinin, LifeAct, EB3, myosin, vimentin, as well as the fusions targeted to various compartments such as clathrin, lysosome-associated membrane protein LAMP1, zyxin (focal adhesions) and mitochondrial signal. (Fig. 2a). The miRFP fusion with histone 2B localized properly in all phases of mitosis and did not affect cell division. Moreover, two spectrally distinct miRFP fusions can be distinguished in the same cell by using two common filter sets (Supplementary Fig. 9).

Next we studied whether miRFP fusions can be imaged beyond the diffraction limit. For this, we applied structured illumination microscopy (SIM) to image miRFP703-tagged α-tubulin and LAMP1 (Fig. 2b,c). SIM technique has allowed a better resolution of α-tubulin filaments than widefield microscopy (Fig. 2b). Almost twice more filaments were detected in a cross-section of the SIM image compared to those visible in the widefield image (Supplementary Fig. 10). The fine circle-like structure of the LAMP1-labelled lysosomes was also visible with SIM (Fig. 2c). miRFPs are good candidates for a crosstalk-free super-resolution imaging with green and red FPs. The three-colour SIM enabled simultaneous visualization of TagGFP2-labelled mitochondria, mCherry-labelled α-tubulin and miRFP703-labelled histone 2B in a cell (Fig. 2d).

### Spectrally distinct reporters for protein-protein interactions

To explore miRFPs in NIR biosensors design, we first applied them to develop monomeric NIR BphP-based BiFC reporters for PPIs. Previous BphP-based complementation reporters were engineered either from dimeric iRFP713 (refs 25, 26) or from weakly dimeric dim IFP1.4 (refs 27, 28). These reporters consist of the PAS domain and the GAF domain fragments fused to two proteins of interest. If the proteins interact, they bring together the PAS and GAF domains, which reconstitute fluorescence.

**Figure 3:**
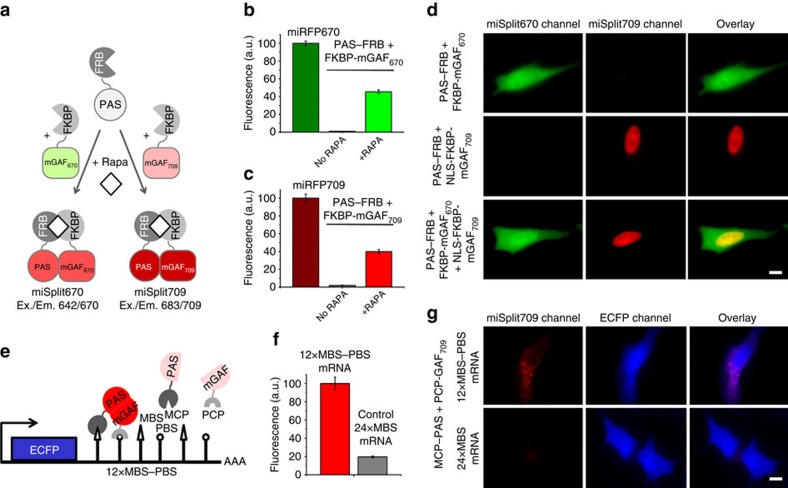
Two bimolecular fluorescence complementation (BiFC) monomeric miSplit reporters. (**a**) Schematics of design and application of miSplit670 and miSplit709 reporters for protein–protein interaction (PPI). The two mSplits share the same PAS fragment that can interact with either mGAF_670_ or mGAF_709_ fragments producing the fluorescence signal corresponding to complemented miSplit670 or miSplit709, respectively. (**b**,**c**) Brightness and complementation contrast of miSplit670 (**b**) and miSplit709 (**c**) in live HeLa cells. HeLa cells were transiently transfected with the plasmids encoding the indicated proteins. Rapamycin (Rapa) was added where indicated. The mean fluorescence intensities of cells were analysed by flow cytometry. Error bars, s.d. (*n*=3; transfection experiments). (**d**) Two-colour imaging of two alternative PPIs in one cell. Transiently transfected HeLa cells expressed cytoplasmic FRB-PAS together with either cytoplasmic FKBP-mGAF_670_ (top) or nuclear FKBP-mGAF_709_ (middle), or both (bottom) of FKBP-fused GAF fragments. Pseudocolour images (miSplit670 channel in green and miSplit709 channel in red) and the overlays are shown. Scale bar, 10 μm. (**e**) Schematics of the approach for NIR low-background RNA imaging. RNA (here mRNA encoding ECFP) is tagged with pairs of RNA-binding motifs, MBS and PBS, which bind bacteriophage coat proteins MS2 (MCP) and PP7 (PCP), respectively. MCP and PCP are fused with two fragments of miSplit reporter. mRNA serves as a scaffold to bring two split fragments together and reconstitute fluorescence. (**f**) mRNA detection with miSplit709. Live HeLa cells co-expressed PAS-MCP and PCP-mGAF_709_ together with ECFP mRNA tagged with 12xMBS-PBS binding sites. ECFP mRNA tagged with 24xMBS binding sites served as a control. The mean fluorescence intensities of cells were analysed by flow cytometry. Error bars, s.d. (*n*=3; transfection experiments). (**g**) Representative images of live HeLa cells analysed in **f**. Pseudocolour images (miSplit709 channel in red and ECFP channel in blue) and the overlay are shown for ECFP mRNA with 12xMBS-PBS (top) and ECFP mRNA with 24xMBS (bottom). Scale bar, 10 μm.

Starting from miRFPs, we engineered two monomeric miSplit reporters (Fig. 3). For this, we cut miRFP670 and miRFP709 between their PAS and GAF domains (Supplementary Fig. 1). The unique feature of the resulting two miSplits, miSplit670 and miSplit709, is that they share the same PAS domain, whereas the GAF domains differ (Fig. 3a). The PAS domain can interact with either mGAF_670_ domain or mGAF_709_ domain, resulting in the miRFP670 or miRFP709 reconstitution, respectively.

We first characterized miSplits using a rapamycin-induced PPI of FRB and FKBP proteins. We fused FRB to the PAS domain and FKBP to either mGAF_670_ or mGAF_709_ domains and tested their effective brightness and BiFC contrast (the ratio between a stimulus-induced fluorescence signal and a fluorescence signal originated from non-specific complementation) in mammalian cells. Both miSplits retained the >40% of effective brightness of parental full-length miRFPs (Fig. 3b,c) and demonstrated the high complementation contrast of >20-fold (Fig. 3b,c). We next studied the BiFC contrast increase in cells on induction of rapamycin-dependent FRB-FKBP interaction. We found that the BiFC contrast of twofold was achieved as early as 30 min after rapamycin addition (Supplementary Fig. 11). Similar to the iRFP713-based BiFC reporters including dimeric iSplit^25^ the complementation of miSplits was irreversible. However, compared with iSplit, both miSplits produced the >4-fold lower background and the substantially higher BiFC contrast in the same conditions (Supplementary Fig. 12). The effective brightness of miSplit670 and miSplit709 was 170% and 60% of the brightness of dimeric iSplit, respectively.

Since the PAS domain is the same for both miSplits we next studied whether both miSplits can be used in the same cell. We added a nuclear localization signal (NLS) to FKBP fused with mGAF_709_ and co-expressed PAS-FRB with either FKBP-mGAF_670_ or NLS-FKBP-mGAF_709_, or with both FKBP fusions (Fig. 3d). We found that similarly to parental miRFP670 and miRFP709, miSplit670 and miSplit709 can be separately imaged in a cell. Thus, miSplits can be applied to discriminate between interaction of one protein fused to the PAS domain with two different proteins fused to the mGAF_670_ and mGAF_709_ domains, respectively.

As a method of RNA detection, BiFC provides lower background of RNA imaging than RNA detection with full-length FPs fused to RNA-binding proteins^29^,^30^. NIR spectra of miSplits should further increase signal-to-background ratio and provide an additional colour for simultaneous imaging of several types of molecules or processes in a cell. To test miSplits for RNA labelling, we chose a system that uses two high affinity RNA-protein interactions, MS2 bacteriophage coat protein (MCP) and its MS2 RNA-binding site (MBS) and PP7 bacteriophage coat protein (PCP) and its PP7 RNA-binding site (PBS)^29^. We fused the MCP and PCP proteins with two fragments of miSplit709 and tagged mRNA encoding an ECFP protein with 12 pairs of MBS-PBS sites (Fig. 3e). As a control, we used ECFP mRNA tagged with 24 copies of MBS only. miSplit709 allowed to specifically visualize the mRNA-12xMBS-PBS molecules in live HeLa cells. The fluorescence signal was >5-fold higher than for mRNA-24xMBS control (Fig. 3f,g). Similar to miSplit709, miSplit670 also enabled the mRNA detection (Supplementary Fig. 13).

### NIR IκBa reporter for imaging of IKK activation

**Figure 4:**
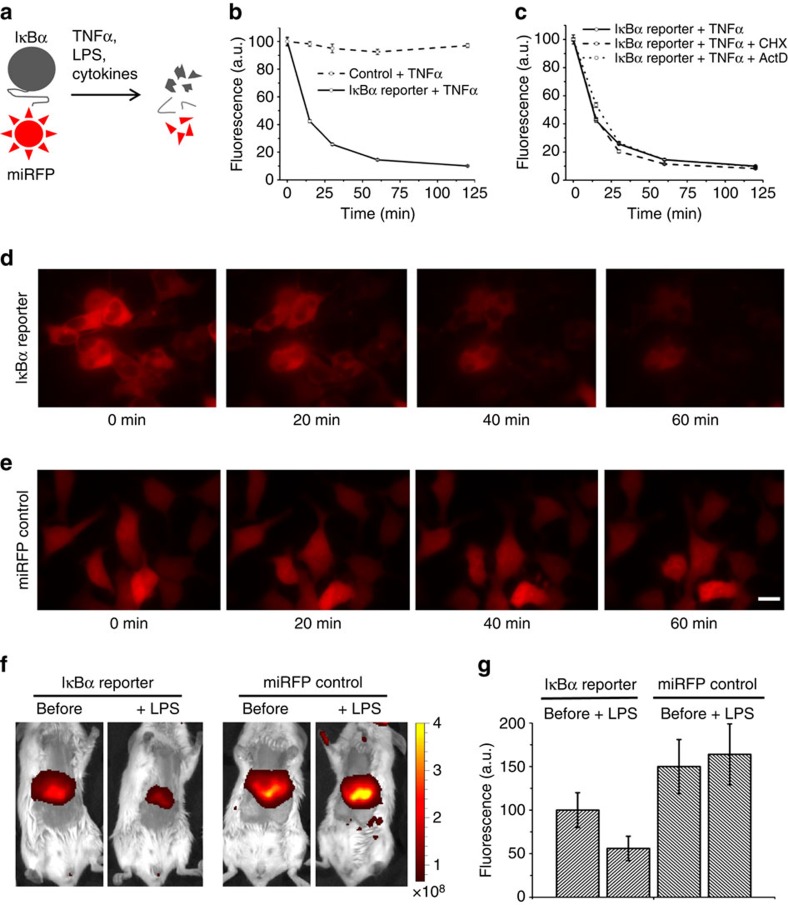
NIR IκBα reporter for canonical NF-κB pathway. (**a**) Schematics showing stimulus-induced degradation of the NIR IκBα reporter. Stimuli inducing IKK activation, such as TNFα, lipopolysaccharide (LPS) and cytokines, lead to IκBα phosphorylation by IKK and degradation of the fusion. (**b**) The response of the NIR IκBα reporter to treatment with TNFα. Live HEK293 cells stably expressing the reporter or untagged miRFP703 control were treated with TNFα. The fluorescence intensity of cells were analysed by flow cytometry at different time points. Error bars, s.d. (*n*=3). (**c**) Effect of pretreatment with translation inhibitor cycloheximide (CHX) or transcription inhibitor actinomycin D (ActD) on the TNFα-induced reporter degradation kinetics studied as in **b**. Error bars, s.d. (*n*=3). (**d**,**e**) Microscopy time-lapse images of live HEK293 cells stably expressing either NIR IκBα-miRFP703 reporter (**d**) or untagged miRFP703 control (**e**) on treatment with TNFα. Scale bar, 10 μm. (**f**) Representative images of a mouse expressing the NIR IκBα reporter in the liver and a control mouse expressing untagged miRFP703 before and 2 h after injection with LPS. The colour bar indicates the total fluorescence radiant efficiency (photons s^−1^ cm^−2^ steradian^−1^ per μW cm^−2^). (**g**) Quantification of the fluorescence changes for the data in **f**. Total radiant efficiencies of the areas corresponding to the livers were quantified. Error bars, s.d. (*n*=3).

We next applied miRFPs to design biosensors based on changes in protein levels in response to activation of specific cellular pathways. By fusing miRFP703 to IκBα, we created a NIR reporter for canonical activation of NF-κB. IκBα is a predominant IκB family member of proteins that inhibit the NF-κB transcription factor, which is a key regulator of immune response, cellular activation, proliferation and apoptosis^31^. Canonical activation of NF-κB depends on induced phosphorylation-dependent IκBα degradation. In resting cells, IκBα sequesters NF-κB dimers in the cytoplasm by masking their NLS. Various stimuli, such as cytokines, TNFα and lipopolysaccharide (LPS), activate IKK kinase, which phosphorylates IκBα marking it as a substrate for polyubiquitination and subsequent degradation by proteasome (Fig. 4a). As a result, NF-κB is released to the nucleus.

To test NIR IκBα reporter in cells, we obtained HEK293 cells stably expressing IκBα-miRFP703. Treatment of these cells with TNFα resulted in a rapid decrease in fluorescence, as expected^32^. In contrast, no changes were observed in control cells expressing unfused miRFP703 (Fig. 4b). An inhibition of the translation and transcription did not affect IκBα-miRFP703 reporter degradation (Fig. 4c). Microscopy of cells stably expressing IκBα-miRFP703 reporter and miRFP703 control (Fig. 4d,e) confirmed the results obtained with flow cytometry and allowed to visualize the cytoplasmic localization of IκBα. If miRFP670 or miRFP709 are fused to IκBα, it is possible to image a second, spectrally resolvable NIR FP (miRFP709 or miRFP670, respectively) simultaneously with the IκBα reporter. We demonstrated this in cells co-expressing IκBα-miRFP670 and nuclear H2B-miRFP709 (Supplementary Fig. 14).

To study IκBα-miRFP reporter *in vivo*, we used an established model of the acute liver inflammation^32^. The gene coding for IκBα-miRFP703 was delivered to a mouse liver by a hydrodynamic gene transfer^33^. We observed that treatment with LPS induced the threefold decrease of the fluorescence signal from the liver (Fig. 4f,g). In control experiment with unfused miRFP703, there was no decrease in the fluorescence signal (Fig. 4f,g).

### NIR cell cycle reporters

**Figure 5:**
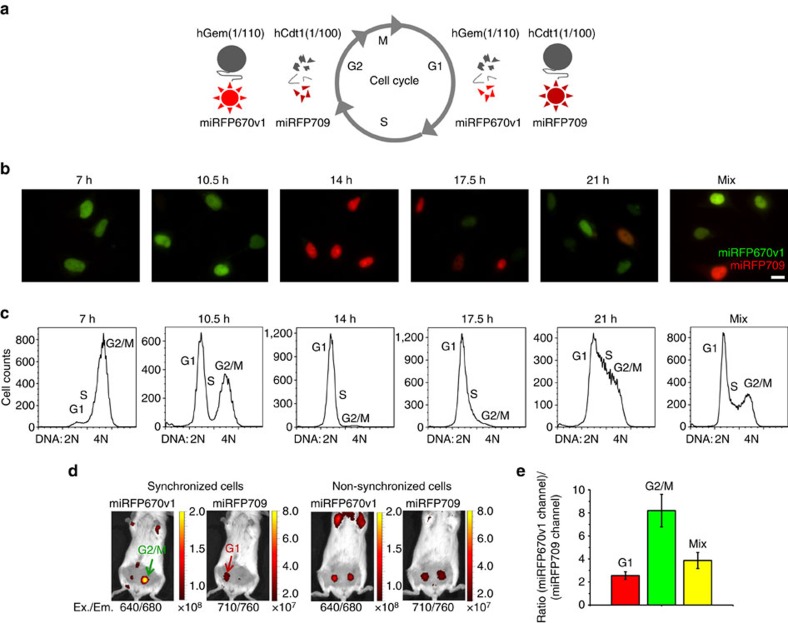
NIR cell cycle reporter based on two spectrally distinct miRFPs. (**a**) Schematics of cell-cycle-dependent fluorescence changes in NIR cell cycle reporter, which consists of a combination of miRFP670v1-hGem(1/110) and miRFP709-hCdt1(1/100) fusions. These fusions are degraded reciprocally during the cell cycle. The miRFP670v1-hGem(1/110) fusion accumulates in S/G2/M phases, whereas the miRFP709-hCdt1(1/100) fusion accumulates in G1 phase. (**b**) Microscopy images of NIR cell cycle reporter in cells at different time points during cell cycle progression. HeLa cells stably expressing NIR cell cycle reporter were released after the synchronization by double thymidine block and analysed at indicated time points. The overlays of two pseudocolour images (miRFP670v1 channel in green and miRFP709 channel in red) are shown. Unsynchronized cells are shown in the most right panel (mix). Scale bar, 10 μm. (**c**) Flow cytometry histograms of Hoechst 33342 fluorescence distribution representing the cell cycle progression for the cells shown in **b**. Cells in **b** and **c** were prepared and analysed in parallel. (**d**) Representative images of mice with implanted cells expressing the NIR cell cycle reporter. A mouse on the left was injected with cells synchronized as in **b** and **c**. The cells in G2/M and G1 phases were injected in the left and right sides of the mouse, respectively. A mouse on the right was injected with the non-synchronized cells into both sides. The two channels for miRFP670v1 and miRFP709 imaging are shown. The colour bars indicate the total fluorescence radiant efficiency (photons s^−1^ cm^−2^ steradian^−1^ per μW cm^−2^). (**e**) The ratios between the fluorescence intensities of the implanted cells in the miRFP670v1 and miRFP709 channels for the data in **d**. Total radiant efficiencies of the areas corresponding to the implanted cells were quantified for each channel and the ratios were calculated. Error bars, s.d. (*n*=6).

We further engineered a cell cycle reporter based on spectrally distinct miRFPs. Available Fucci (fluorescence ubiquitination-based cell cycle indicator) cell cycle reporters are based on green and red GFP-like FPs fused to cell cycle regulated proteins, Geminin and Cdt1, which are involved in licensing of replication origins^34^,^35^. Fucci reporter offers an accurate and versatile visualization of the cell cycle progression and facilitates studies of developmental processes, such as pattern formation, morphogenesis, cell differentiation, growth, cell migration and cell death^36^. To design a NIR cell cycle reporter, which will allow non-invasive monitoring of cell cycle in both cells and whole organisms, we used the same approach but applied miRFP670v1 and miRFP709 (Fig. 5a). Two specific E3 ligase activities mark Geminin and Cdt1 with ubiquitin for proteosomal degradation in a cell-cycle-dependent manner. As a result, protein levels of Geminin and Cdt1 oscillate in antiphase. The Cdt1 level is the highest during G1 phase, and then it degrades in S/G2/M phases, whereas the Geminin level is the highest during S/G2/M phases, and it degrades in G1 phase. Thus, the levels of the fused FPs also undergo reciprocal changes, resulting in the dynamic colour change during cell cycle progression.

To find an optimal reporter, we created miRFP670v1 and miRFP709 fusions with different deletion mutants of Cdt1 and Geminin: hCdt1(30/120)^34^, hCdt1(1/100)^37^, hGem(1/110)^34^ and hGem(1/60)^35^. The truncated Cdt1 and Geminin variants are used to not perturb the normal cell cycle^34^. We tested their combinations for specificity to cell cycle phases and possible interference with the cell cycle. We selected a combination of miRFP670v1-hGem(1/110) and miRFP709-hCdt1(1/100). HeLa cells stably expressing these two fusions, which we call NIR cell cycle reporter, divided normally for many generations.

To test the performance of NIR cell cycle reporter, we synchronized division of HeLa cells stably expressing this reporter using a double thymidine block procedure^38^. We analysed the cells by flow cytometry and fluorescence microscopy at different time points after release from the block. To analyse the cell cycle phase, cells were labelled with Hoechst 33342, whose fluorescence signal is proportional to the DNA content and enables to reveal the various phases of the cell cycle. For microscopy imaging of the reporter, we used two filter sets allowed to distinguish miRFP670 and miRFP709 as above. The two miRFPs are also distinguishable by flow cytometry (Supplementary Fig. 15a–d). Thus, NIR cell cycle reporter can be studied by both microscopy and flow cytometry. As expected, the changes in Hoechst 33342 fluorescence corresponded to the reciprocal changes in the levels of the miRFP670v1 and miRFP709 fusions during the cell cycle (Fig. 5b,c). Indeed, in the G2/M phase (7 h after a release from the cell cycle arrest) the cells predominantly expressed miRFP670v1 as can be seen by microscopy (Fig. 5b,c) and flow cytometry (Supplementary Fig. 15e). In contrast, in G1 phase (14 h after a release from the cell cycle block), the cells expressed miRFP709 (Fig. 5b,c, Supplementary Fig. 15e). As expected, the non-synchronized cells expressed either miRFP670v1, or miRFP709 or both. We also tested and found that NIR cell cycle reporter works in HEK293 cells, similar to HeLa cells (Supplementary Fig. 16).

To test whether NIR cell cycle reporter can be used to discriminate between cells in G1 phase and in G2/M phase *in vivo*, we synchronized cells as above. Then, we injected the cells in G1 phase in the left mammary gland and the cells in the G2/M phase in the right mammary gland of mice (Fig. 5d). Imaging of mice in two different channels allowed distinguishing between cells in G1 and G2/M phases. A ratio between the fluorescence signals in these channels reflected the proliferation status of cells (Fig. 5e). For injected non-synchronized cells, this ratio lay between the two ratios obtained for cells synchronized in G1 and G2/M phases (Fig. 5d,e). We also found that this ratio remained approximately the same when the injected cells had grown into tumours (Supplementary Fig. 17).

## Discussion

Monomeric state of three miRFPs and their high effective brightness in mammalian cells without supply of external BV or co-expression of BV-producing HO make them advanced NIR protein tags (Table 1). Their protein fusions exhibit proper localization in cellular filaments and intracellular compartments (Fig. 2a), while the most spectrally distant miRFP670 and miRFP709 enable two-colour protein labelling (Supplementary Fig. 9). Super-resolution SIM imaging with miRFP fusions allows visualization of the finer details of organelles and filaments than conventional widefield microscopy (Fig. 2b,c). miRFPs enabled crosstalk-free three-colour microscopy together with conventional green and red FPs, as we demonstrated in three-colour SIM (Fig. 2d).

Importantly, miRFPs substantially outperform the only other monomeric NIR FP, mIFP, in terms of the effective brightness (Supplementary Fig. 5) and the photostability in mammalian cells (Supplementary Fig. 2e). Yet another advantage of mRFPs is availability of spectrally resolvable variants, which can be combined for multicolour NIR imaging.

Moreover, bright multicolour miRFPs now enable development of a wide range of NIR reporters and biosensors for various intracellular processes involving interaction of proteins, signalling cascades and cell fate, among others. Here we applied miRFPs to design just a few of them, such as reporters for PPI, for RNA, for NF-κB pathway and for cell cycle progression.

miSplit670 and miSplit709 are the first NIR BiFC reporters that are based on truly monomeric NIR FPs. Both miSplits are comparable in brightness to dimeric iSplit^1^, but have much lower non-specific BiFC background and substantially higher BiFC contrast (Supplementary Fig. 12). In contrast to dimeric iSplit, miSplits can be applied for screening of novel PPIs because the monomeric state of miSplit parts will not interfere with PPIs themselves. Furthermore, the combination of spectrally distinct miSplits enables to distinguish PPIs of one protein with two alternative partners (Fig. 3a,d).

The monomeric nature and low complementation background of miSplits allow NIR RNA imaging (Fig. 3e–g and Supplementary Fig. 13). Since miSplits are monomeric, all individual mRNA molecules are detected separately in a cell. The fluorescence level of the miSplit BiFC reports on the amount of transcribed mRNA, while the fluorescence pattern reports on the mRNA intracellular localization. In contrast to other RNA labels, the NIR miSplit labelling is suitable for RNA imaging both in cells and *in vivo*.

Similar to GFP-based BiFC split reporters, miSplits are irreversible. The BiFC irreversibility enables integration, accumulation and subsequent detection of transient PPIs and low affinity complexes^39^,^40^. In contrast to reversible luciferase split reporters, BiFC reporters (i) allow subcellular localization of a PPI by microscopy, (ii) can be applied for PPI screening, (iii) can be applied in multicolour detection of several PPIs and (iv) do not require supply of substrate^41^. Similarly to GFP-based BiFC split pairs, miSplits should enable monitoring of activities of drug targets, such as GPCR and RTK receptors^40^, identifying of potential off-target effects of drug candidates by detection of downstream PPIs associated with specific signalling pathways^40^, and genome-wide PPI studies^42^. NIR fluorescence of miSplit reporters enable their use for non-invasive *in vivo* imaging and as additional colours for detection of several PPIs.

The NIR IκBα reporter allows non-invasive studies of canonical activation of NF-κB pathway in cells and in animal tissues (Fig. 4). Compared with luciferase-based IκBα reporter^32^, NIR IκBα reporter is suitable for longitudinal quantitative microscopy in live cells and *in vivo* imaging without substrate injection. Our data show that a fluorescence biosensor, such as IκBα reporter, does not always require a second reference colour, since its signal is stable over time without stimuli (Fig. 4d,e; Supplementary Fig. 14). In contrast, a reference signal is necessary for luciferase-based reporters, since bioluminescence changes over time as the result of time-dependent substrate delivery, substrate consumption and degradation. The NIR IκBα reporter could be applied to study pharmacodynamics of ligands and drugs, which target NF-κB signalling. IKK-dependent activation of NF-kB pathway is a promising target for drug development since it is involved in chronic inflammation conditions, such as inflammatory bowel disease, asthma, rheumatoid arthritis^43^,^44^ and cancer^45^. The NIR IκBα reporter is an example of a biosensor based on the post-translational changes in protein levels. Analogous reporters for other signalling pathways can be created with bright miRFPs by using the same approach.

The NIR cell cycle reporter relies on two spectrally resolvable miRFPs, whose fusions accumulate reciprocally during the cell cycle. We demonstrated that this reporter can be applied in cells with analysis by microscopy or by flow cytometry and also in non-invasive whole-body studies by spectral imaging (Fig. 5, Supplementary Figs 15–17). The ratio between signals of two miRFPs serves as an indicator of proliferation status of the cell population *in vivo*. In contrast to green-red GFP-based Fucci indicator, NIR cell cycle reporter is suitable for non-invasive *in vivo* studies.

In conclusion, the developed spectrally distinct miRFPs and miRFP-based biosensors allow non-invasive multicolour visualization of biological processes across scales, from super-resolution microscopy to tissue and whole-body animal imaging. The ability to use the same probe at the cellular and organismal levels will advance studies of cancer, neuroscience, immunology, developmental and stem cell biology, as well as will make preclinical drug screening significantly faster and more efficient.

## Methods

### Mutagenesis and directed molecular evolution

For expression in bacteria, the PAS-GAF domains (first 315 amino acids) of *Rp*BphP1 from *Rhodopseudomonas palustris* was cloned into pBAD/His-B vector (Life Technologies/Invitrogen). Site-specific mutagenesis was performed with QuikChange mutagenesis kit (Agilent Technologies), random mutations were introduced using GeneMorph II random mutagenesis kit (Agilent Technologies). LMG194 host cells (Invitrogen) were used for protein expression. A pWA23h plasmid encoding HO from *Bradyrhizobium ORS278* (hmuO) under the rhamnose promoter was co-transformed with a pBAD/His-B plasmid encoding a fluorescent protein^16^,^46^. Libraries of mutants consisted of >10^6^ independent clones. Bacterial cells were incubated overnight at 37 °C in RM minimal medium with ampicillin and kanamycin. To start protein expression, 0.002% arabinose and 0.02% rhamnose were added. After growing for 6–8 h at 37 °C, the cells were incubated at 18 °C for 24 h. Flow cytometry screening was performed on MoFlo XDP (Beckman Coulter) fluorescence-activated cell sorter. 647 nm laser for excitation and a 700 nm LP emission filter were used for positive selection of fluorescent cells. Before sorting, bacterial pellet was washed with phosphate buffered saline (PBS) and diluted to an optical density of 0.03 at 600 nm. Collected cells were rescued in SOC medium at 37 °C for 1 h and then grown on LB/ampicillin/kanamycin Petri dishes supplemented with 0.02% arabinose and 0.2% rhamnose. IVIS Spectrum imager (PerkinElmer/Caliper) was used for screening of spectrally distinct mutants on Petri dishes. Data were analysed with the LivingImage v.4.3.1 software (PerkinElmer/Caliper).

The mutants selected in bacteria were tested for the oligomeric state by size exclusion chromatography as described below. We also tested their brightness in HeLa cells transfected with plasmids obtained after the FP genes were swapped with a gene encoding EGFP in the pEGFP-N1 plasmid (Clontech). A mixture of several selected mutants was then used as a template for the next round of mutagenesis.

### Protein expression and characterization

Protein expression in bacteria was performed as described above for sorting of libraries of mutants. Specifically, at the first step HO was expressed with addition of 0.02% rhamnose for 5 h at 37 °C. Then expression of a fluorescent protein was induced by addition of 0.002% arabinose for 12 h at 37 °C following by 24 h at 18 °C. In addition to LMG194, TOP10 bacterial cells (Life Technologies/Invitrogen) bearing the pWA23h plasmid were used as a host. Proteins were purified with Ni-NTA agarose (Qiagen). Proteins were eluted with PBS containing 100 mM EDTA, instead of imidazole. Then the samples were desalted using PD-10 desalting columns (GE Healthcare).

FluoroMax-3 spectrofluorometer (Jobin Yvon) was used for recording of fluorescence spectra, Hitachi U-2000 spectrophotometer was used for absorbance measurements. To determine extinction coefficient, we calculated a ratio between the maximum absorbance of the main peak at Q band and the side peak at Soret band and assumed that extinction coefficient at Soret band corresponds to 39,900 M^−1^ cm^−1^ (refs 3, 4). To determine quantum yield, we measured fluorescence signal of a purified protein in parallel with an equally absorbing Nile blue dye (quantum yield is 0.27 in an acidic ethanol^47^) and compared the signal at several dilutions. pH titrations were done using a series of buffers (100 mM sodium acetate, 300 mM NaCl for pH 2.5–5.0 and 100 mM NaH_2_PO_4_, 300 mM NaCl for pH 4.5–9.0).

To perform size exclusion liquid chromatography, 2 ml volumes of the purified miRFPs, iRFP713 or IFP2.0 samples were applied on the HiLoad 16/600 Superdex 200 column (GE Healthcare) equilibrated with 20 mM Hepes buffer pH 7.4 containing 0.1 mM EDTA, 10% glycerol, 150 mM NaCl, 0.2 mM phenylmethane sulfonyl fluoride (PMSF), 0.01% NP-40 and 0.5 mM Benzamidin. A 1 ml min^−1^ flow rate was used. The proteins were also analysed in 20 mM Tris-HCl pH 8.0, 200 mM NaCl, 1 mM PMSF with the same result. The column was calibrated using the gel filtration standards (Bio-Rad Laboratories).

Analytical ultracentrifugation was conducted at 20 °C and 58,000 r.p.m. with an Optima XL-I centrifuge (Beckman Coulter) using the AN-60Ti rotor and the absorption optics set to 645 nm. Sednterp v.20120828beta software was used to calculate the partial specific volume of the proteins from their sequence and the density and viscosity of the buffers. The sedimentation parameters were corrected to standard conditions (20,w) using these values. For sedimentation velocity (SV) experiments, 350 μl of protein sample and an equal volume of PBS buffer were loaded into two-sector cell assemblies with the protein concentration corresponding to A_645_ ≈0.9. Fifty scans were collected over the course of a centrifuge run. A subset of scans, beginning with those where a clear plateau was evident between the meniscus and the boundary, was selected for time-derivative analysis using DCDT+ v.2.4.2 software^48^,^49^.

### Construction of mammalian plasmids

To construct mammalian expression plasmids, the respective genes of FPs were PCR-amplified as AgeI-NotI fragments and swapped with a gene encoding EGFP in the pEGFP-N1 plasmid (Clontech). IFP2.0-N1 and mIFP-N1 plasmids were acquired from Addgene (#54785 and #54620, respectively).

For protein tagging and labelling of intracellular structures study, miRFPs were amplified, digested with restriction enzymes and then swapped with mTagBFP2 either as C- (for α-tubulin and clathrin) or N-terminal fusions (for keratin, α-actinin, LifeAct, EB3, myosin, vimentin, clathrin, LAMP1, zyxin, H2B and mitochondrial signal) as previously described^50^. C-terminal fusions (SGGGG)_n_ linker was increased to 30 amino acids. N-terminal fusions linker length was left unchanged.

To create an IκBα reporter plasmid (CMV-IκBα-miRFP703), we used a CMV-IκBα-FLuc plasmid kindly provided by S. Achilefu and D. Piwnica-Worms. A FLuc gene was replaced with one of the miRFP genes. Kozak sequence was deleted in the CMV-IκBα-miRFP703 and CMV-miRFP control plasmids.

miSplit670 and miSplit709 reporter plasmids, which are pC4-RHE-PAS, pC4EN-F1-mGAF670 and pC4EN-F1-mGAF709, were constructed from an iSplit plasmids^25^ by swapping either PAS or GAF domains. A linker -ggggsggggs- was left unchanged. Where appropriate, an NLS sequence in the pC4EN-F1 plasmid was deleted by site-directed mutagenesis.

For mRNA labelling, a CMV-PAS-MCP plasmid was constructed as follows. PAS-ggggsggggs- without STOP codon was amplified as a single fragment and inserted into the C1 vector backbone using AgeI and KpnI sites, MCP was amplified from an ubc-nls-ha-MCP-VenusN-nls-ha-PCP-VenusC plasmid (Addgene, #52985) and inserted at KpnI and BamHI sites. The cmv-PCP-mGAF670 and cmv-PCP-mGAF709 plasmids were constructed as follows. A PCP without STOP codon was amplified from an ubc-nls-ha-MCP-VenusN-nls-ha-PCP-VenusC plasmid and then inserted into the C1 vector backbone using AgeI and EcoRI restriction sites. A -ggggsggggs-miGAF was amplified as a single fragment and inserted using EcoRI and KpnI sites. A phage-cmv-cfp-12xMBS-PBS was obtained by swapping a 12xMBS-PBS fragment from a Pcr4-12xMBS-PBS (Addgene, #52984) with 24xMS2 in a phage-cmv-cfp-24xms2 plasmid (Addgene, #40651). An ubc-nls-ha-MCP-VenusN-nls-ha-PCP-VenusC, a phage-cmv-cfp-24xMS2 and a Pcr4-12xMBS-PBS plasmids were gifts from B. Wu and R. Singer.

Plasmids encoding several green-red Fucci cell cycle reporters were provided by A. Miyawaki. The mKO2 and mAG genes fused with hCdt1(30–120), hCdt1(1/100), hGem(1/110) and hGem(1/60) sequences in the pCSII-EF-MCS plasmids were swapped with the miRFP709 or miRFP670v1 genes.

### Mammalian cells and transfection

HeLa, HEK293, U87, U2OS and Cos-1 cells were purchased from the ATCC and were not additionally authenticated or tested for mycoplasma contamination. HeLa cells were grown in a DMEM medium supplemented with 10% fetal bovine serum 0.5% penicillin–streptomycin and 2 mM glutamine (Life Technologies/Invitrogen). HEK293, U87, U2OS and Cos-1 cells were grown in the same medium as the HeLa cells. For microscopy, cells were cultured in 35 mm glass-bottom Petri dishes with no. 1 coverglass (MatTek).

Plasmid transfections were performed using an Effectene reagent (Qiagen). Stably expressing cells were selected with 0.7 mg ml^−1^ G418 antibiotic. Sorting of positive cells was performed using a FACSAria sorter (Beckman Coulter) equipped with a 635 nm laser and a 680 nm LP emission filter.

NIR cell cycle reporter was delivered by cotransduction with lentiviruses. Replication-defective self-inactivating lentivirus vectors were used. Fusions miRFP709 with hCdt1(1/100) or hCdt1(30/120) and miRFP670v1 with hGem(1/110) or hGem(1/60) were cloned into pCSII-EF-MCS vector^51^. Lentiviral particles were packaged as described^52^ using a plasmid set pCMV-GagPol, pCMV-REV and pVSV-g kindly provided by P. Chumakov and pCSII-EF-MCS plasmids containing fusions co-transfected in HEK293T cells. For infection of target cells, viral preparations were diluted in complete growth media supplemented with 4 μg ml^−1^ Polybrene.

Primary cultures of hippocampal neurons were isolated as described^53^. Cells were transfected with a Lipofectamine 2000 (Life Technologies/Invitrogen) and imaged 72 h after the transfection

### Widefield and super-resolution microscopy

Life HeLa cells were imaged on Olympus IX81 inverted epifluorescence microscope 48 h after transfection. The microscope is equipped with a 200 W metal halide arc lamp (Lumen220 Pro; Prior), 100 × 1.4 numerical aperture (NA) oil immersion objective lens (UPlanSApo; Olympus). To separately image miRFP670 and miRFP709 in one cell (two NIR colour imaging), the two filter sets (605/40 nm exciter and 667/30 nm emitter, and 682/12 nm exciter and 721/42 nm emitter) (Chroma) were used. For one NIR colour, a Cy5.5. filter set (665/45 nm exciter and 725/50 nm emitter) was used. The microscope was operated with SlideBook v.4.1 software (Intelligent Imaging Innovations).

To determine photostability, proteins were transiently expressed in HeLa cells and imaged at determined time periods, as described above. Obtained raw data were normalized to corresponding absorbance spectra and extinction coefficients of the proteins, the spectrum of 200 W Me-Ha arc lamp and the transmission of 665/45 nm photobleaching filter.

For SIM imaging, cells were fixed with 2% (w/v) paraformaldehyde for 15 min and mounted in Prolong Gold. Multichannel SIM images were acquired using a Nikon Structured Illumination system on an inverted Nikon ECLIPSE Ti-E equipped with a 100 × 1.49 NA oil immersion objective lens. Multicolour fluorescence was generated using diode lasers (405, 488 and 647 nm). Acquisition was performed with electron-multiplying CCD cameras (Andor iXon3 DU897) of 512 × 512 pixel frame size. Three reconstruction parameters (illumination modulation contrast, high-resolution noise suppression and out of focus blur suppression) were extensively tested to generate consistent images across experiments without abnormal features or artifacts and producing the best Fourier transforms. The images were processed using Nikon Elements software.

### Flow cytometry and reporter characterization in cells

Flow cytometry analysis was performed using a BD LSRII flow cytometer equipped with the 355, 488, 561 and 640 nm lasers and a set of emission filters. 20,000–50,000 events for each cell type were analysed. To quantify cell fluorescence, a mean fluorescent intensity of the double-positive population in the NIR channel was divided by a mean fluorescence intensity of the same population in the green channel, thus normalizing NIR signal to transfection efficiency.

All the observations for miSplit reporters were performed in HeLa cells 46–48 h after transfection. Where needed, 100 nM rapamycin was added 24 h before observation. Fluorescence was quantified using flow cytometry or fluorescence microscopy.

For IκBα reporter studies, preclonal mixtures of HEK293 cells stably expressing IκBα-miRFP or miRFP control were treated with TNFα (20 ng ml^−1^; Sigma-Aldrich) at indicated time points before flow cytometry. Cycloheximide (100 mg ml^−1^; Sigma-Aldrich) or actinomycin D (1 μM; Sigma-Aldrich) were added 1 h beforeto the TNFα treatment.

For NIR cell cycle reporters studies by flow cytometry, microscopy and *in vivo* experiments, cells were synchronized by double thymidine block-release procedure as described in^38^,^54^. To quantify DNA content, DNA was labelled with 5 μg ml^−1^ of Hoechst 33342 (BD Biosciences) for 30 min before flow cytometry.

### Characterization of reporters in mice

Mice were fed with AIN-93M Maintenance Purified Diet (TestDiet) to reduce the intrinsic autofluorescence level. For imaging belly fur was removed using a depilatory cream. All animal experiments were performed in an AAALAC approved facility using protocols approved by the Albert Einstein College of Medicine Animal Usage Committee.

For IκBα reporter characterization, *in vivo* transfection of mouse hepatocytes was performed using the hydrodynamic method as described previously^33^. Briefly, IκBα–miRFP703 (20 μg) or miRFP703 control plasmids (20 μg) were diluted in PBS in a volume of 1 ml per 10 g body weight and injected rapidly (5–7 s) into tail veins of mice using a 3 ml syringe fitted with a 27G needle. The 5–7-week-old female FVB mice (National Cancer Institute, NIH) were used. Twenty-four hours later, mice were anaesthetized (isoflurane) and imaged for liver miRFP expression using the IVIS Spectrum system using 675/30 nm excitation and 720/20 nm emission filter set. Immediately following this pretreatment imaging, LPS (4 μg g^−1^ body weight, intravenously) was administered. Post-treatment imaging was performed as above, 2 h later. To quantify fluorescence signals, regions of interest were defined manually over the liver. The fluorescence signals of the same regions of interests in untreated mice were considered as a background and subtracted.

For NIR cell cycle reporter characterization, 10^7^ cells expressing the reporter were subcutaneously implanted into the mammary gland of SCID/NCr mice (female, 5–7 weeks old) (Taconic) and imaged 2 h later using the IVIS Spectrum. Background-subtracted images of total radiant efficiencies of the regions corresponding to cells or tumours were used for quantification. Filter channels used for calculation of the ratio between miRFP670v1 and miRFP709 signals were 640/30 nm excitation and 680/20 nm emission; and 710 nm excitation and 760 nm emission. The tumours were excised postmortem and imaged using the IVIS Spectrum. All quantitative analysis of fluorescence signals was performed using the Living Image software (PerkinElmer/Caliper).

### Data availability

The data that support the findings of this study are available from the corresponding author on request. Sequences of the reported proteins are deposited in the Genbank database with the following accession numbers: miRFP670, KX421097; miRFP703, KX421098; and miRFP709, KX421099.

## Additional information

**How to cite this article:** Shcherbakova, D. M. *et al.* Bright monomeric near-infrared fluorescent proteins as tags and biosensors for multiscale imaging. *Nat. Commun.* 7:12405 doi: 10.1038/ncomms12405(2016).

## Supplementary Material

Supplementary Information Supplementary figures 1-17

## Figures and Tables

**Table 1:** Selected NIR FPs engineered from bacterial phytochromes with the demonstrated *in vivo* applications.

NIR FP	Ex, nm	Em, nm	Extinction coefficient, M^−1^ cm^−1^	Quantum yield, %	Molecular brightness versus iRFP713, %	Oligomeric state	Photostability in mammalian cells, t_1/2_, s	p*K*a	Brightness in HeLa cells versus iRFP713, %*	Ref.
miRFP670_v1_	644	670	71,300±2,100	11.6±0.5	134	Monomer	155±6	4.5	30.0±1.7	This work
miRFP670	642	670	87,400±2,600	14.0±0.6	198		155±5	4.5	72.0±3.5	
miRFP703	674	703	90,900±2,700	8.6±0.5	127		394±7	4.5	37.4±2.4	
miRFP709	683	709	78,400±2,400	5.4±0.2	69		192±5	4.5	30.1±1.9	
mIFP†	683 (683)	705 (704)	65,900±2,000 (82,000)	6.9±0.9 (8.4)	74	Monomer	54±5	4.5	14.5±2.9	1717
IFP2.0†‡	688 (690)	709 (711)	72,900±2,200 (98,000)	6.8±0.2 (8.1)	80	Dimer§	108±6	4.5	7.9±1.1	1818
iRFP670	643	670	114,000	12.2	225	Dimer	290	4.0	119	1616
iRFP682	663	682	90,000	11.1	162		490	4.5	105	
iRFP702	673	702	93,000	8.2	124		630	4.5	61	
iRFP713 (aka iRFP)	690	713	98,000	6.3	100		960	4.5	100	44
iRFP720	702	720	96,000	6.0	93		490	4.5	110	1616

FP, fluorescent proteins; NIR, near-infrared. Error, s.d. (*n*=3).

^*^Determined as effective NIR fluorescence in transiently transfected live HeLa cells with no supply of exogenous BV and after normalization to fluorescence of co-transfected EGFP.

^†^Characteristics of IFP1.4, IFP2.0 and mIFP from the original paper^17^ are shown in parentheses. Parameters determined in this paper are shown without parentheses.

^‡^Only IFP2.0 is presented as the latest version in a series of the IFP proteins^18^.

^§^Although IFP2.0 was originally reported as a monomer^18^, later it was found to be a dimer^1^, which was also confirmed by its developers^17^.
