# Supplementary material for: Bright monomeric near-infrared fluorescent proteins as tags and biosensors for multiscale imaging
Source: Nat Commun. 2016 Aug 19;7:12405. doi: 10.1038/ncomms12405 (PMC4992171; doi:10.1038/ncomms12405)
Supplement: Supplementary Information — Supplementary figures 1-17 [file ncomms12405-s1.pdf]

Supplementary Figure 1. Alignment of the amino acid sequences of miRFPs with parental *Rp*BphP1 bacterial phytochrome.

```
RpBphP1      MVAGHASGSPAFGTADLSNCEREEIHLAGSIQPHGALLVVSEPDHRIIQASANAAEFLN
miRFP670v1   MVAGHASGSPAFGTASHSNSEHEEIHLAGSIQPHGALLVVSEHDHRVIQASANAAEFLN
miRFP670     MVAGHASGSPAFGTASHSNCEHEEIHLAGSIQPHGALLVVSEHDHRVIQASANAAEFLN
miRFP703     MVAGHASGSPAFGTASHSNCEHEEIHLAGSIQPHGALLVVSEHDHRVIQASANAAEFLN
miRFP709     MVAGHASGSPAFGTASHSNCEHEEIHLAGSIQPHGALLVVSEHDHRVIQASANAAEFLN

RpBphP1      LGSVLGVPLAEIDGDLLIKILPHLDPTAEGMPVAVRCRIGNPSTEYDGLMHRPPEGGLIIE
miRFP670v1   LGSVLGVPLAEIDGDLLIKILPHLDPTAEGMPVAVRCRIGNPSTEYCGLMHRPPEGGLIIE
miRFP670     LGSVLGVPLAEIDGDLLIKILPHLDPTAEGMPVAVRCRIGNPSTEYCGLMHRPPEGGLIIE
miRFP703     LGSVLGVPLAEIDGDLLIKILPHLDPTAEGMPVAVRCRIGNPSTEYCGLMHRPPEGGLIIE
miRFP709     LGSVLGVPLAEIDGDLLIKILPHLDPTAEGMPVAVRCRIGNPSTEYCGLMHRPPEGGLIIE

RpBphP1      LERAGPPIDLSGTLAPALERIRTAGSLRALCDDTALLFQQCTGYDRVMVYRFDEQGHGE
miRFP670v1   LERAGPSIDLSGTLAPALERIRTAGSLRALCDDTVLLFQQCTGYDRVMVYRFDEQGHGL
miRFP670     LERAGPSIDLSGTLAPALERIRTAGSLRALCDDTVLLFQQCTGYDRVMVYRFDEQGHGL
miRFP703     LERAGPSIDLSGTLAPALERIRTAGSLRALCDDTVLLFQQCTGYDRVMVYRFDEQGHGL
miRFP709     LERAGPSIDLSGTLAPALERIRTAGSLRALCDDTVLLFQQCTGYDRVMVYRFDEQGHGL

RpBphP1      VFSERHVPGLESYFGNRYPSSDIPQMARRLYERQRVRVLVDVSYQPVPLEPRLSPLTGR
miRFP670v1   VFSECHVPGLESYFGNRYPSSTVPQMARQLYVRQRVRVLVDVTYQPVPLEPRLSPLTGR
miRFP670     VFSECHVPGLESYFGNRYPSSTVPQMARQLYVRQRVRVLVDVTYQPVPLEPRLSPLTGR
miRFP703     VFSECHVPGLESYFGNRYPSSLVPQMARQLYVRQRVRVLVDVTYQPVPLEPRLSPLTGR
miRFP709     VFSECHVPGLESYFGNRYPSSFIPQMARQLYVRQRVRVLVDVTYQPVPLEPRLSPLTGR

RpBphP1L     DLDMSGCFLRSMSPIHLQYLKNMGVRATLVVSLVVGGKLWGLVACHHYLPRFIHFELR
miRFP670v1   DLDMSGCFLRSMSPCHLQFLKDMGVRATLAVSLVVGGKLWGLVVCHHYLPRFIRFELR
miRFP670     DLDMSGCFLRSMSPCHLQFLKDMGVRATLAVSLVVGGKLWGLVVCHHYLPRFIRFELR
miRFP703     DLDMSGCFLRSMSPIHLQFLKDMGVRATLAVSLVVGGKLWGLVVCHHYLPRFIRFELR
miRFP709     DLDMSGCFLRSMSPIHLQFLKDMGVRATLAVSLVVGGKLWGLVVCHHYLPRFIRFELR

RpBphP1     AICELLAEAIATRITALES-
miRFP670v1  AICKRLAERIATRITALES
miRFP670    AICKRLAERIATRITALES
miRFP703    AICKRLAERIATRITALES
miRFP709    AICKRLAERIATRITALES
```

Amino acid sequences of engineered miRFP670v1, miRFP670, miRFP703 and miRFP709 aligned with that of the wild-type PAS-GAF domains of parental *Rp*BphP1. The amino acid substitutions in the miRFP proteins are highlighted in yellow. To create miSplit reporters, the miRFP670 and iRFP709 sequences were cut between the PAS and GAF domains. Four amino acid residues present in both the PAS fragment and the GAF fragment of mSplits are highlighted in green.

Supplementary Figure 2. Biochemical and photochemical properties of miRFPs.

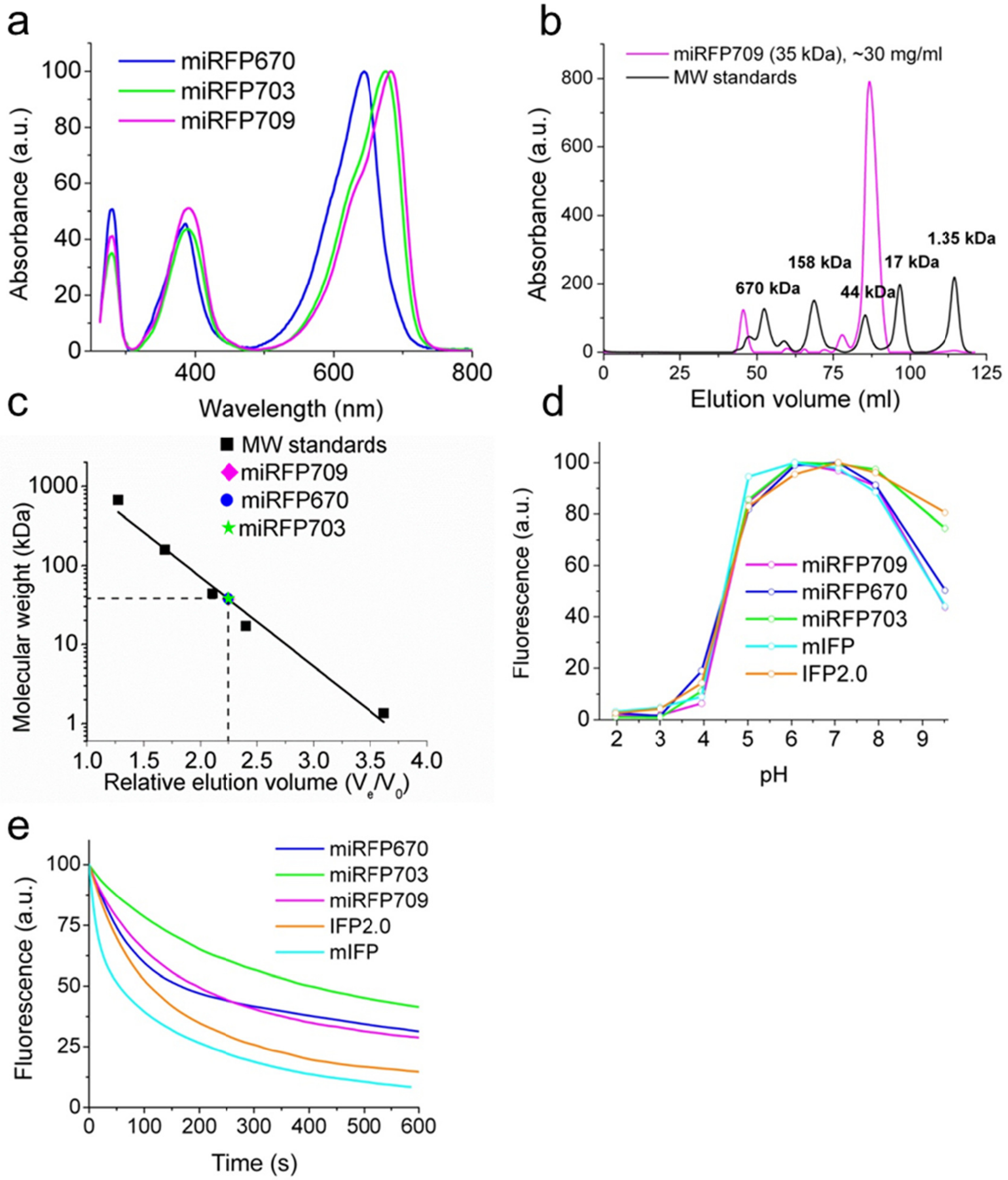

(a) Absorbance spectra of miRFPs. **(b)** Size exclusion chromatography of the miRFP709 protein at concentration of 30 mg ml^{-1} and the molecular weight standards. **(c)** Size exclusion chromatography calibration plot. V_e, elution volume; V_o, void volume of the column. **(d)** pH dependencies of NIR fluorescence for several NIR FPs. **(e)** Photobleaching in live HeLa cells. The curves were normalized to absorbance spectra and extinction coefficients of NIR FPs, spectrum of the lamp, and transmission of the excitation filter.

Supplementary Figure 3. Size exclusion chromatography analysis of NIR FPs.

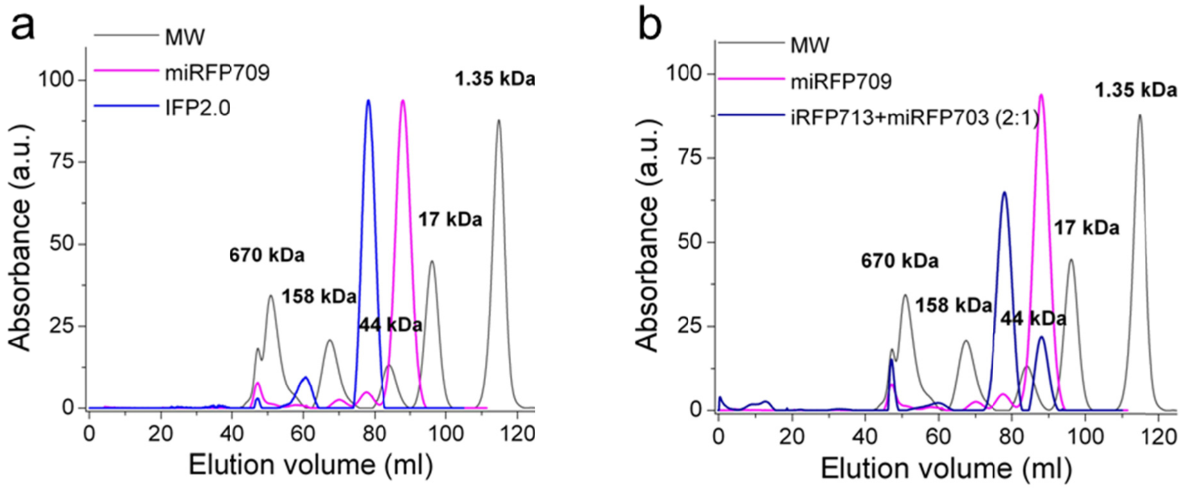

(a) Size exclusion chromatography of the miRFP709 and IFP2.0 proteins at concentration of 1 mg ml^{-1} and the molecular weight standards. The samples were run in the same conditions and then overlaid. **(b)** Size exclusion chromatography of the mixture of iRFP713 and miRFP703 proteins (in a ratio of 2:1; total protein concentration 1.5 mg ml^{-1}) overlaid with the miRFP709 protein at 1 mg ml^{-1} and the molecular weight standards run in the same conditions.

Supplementary Figure 4. Analytical ultracentrifugation analysis of NIR FPs.

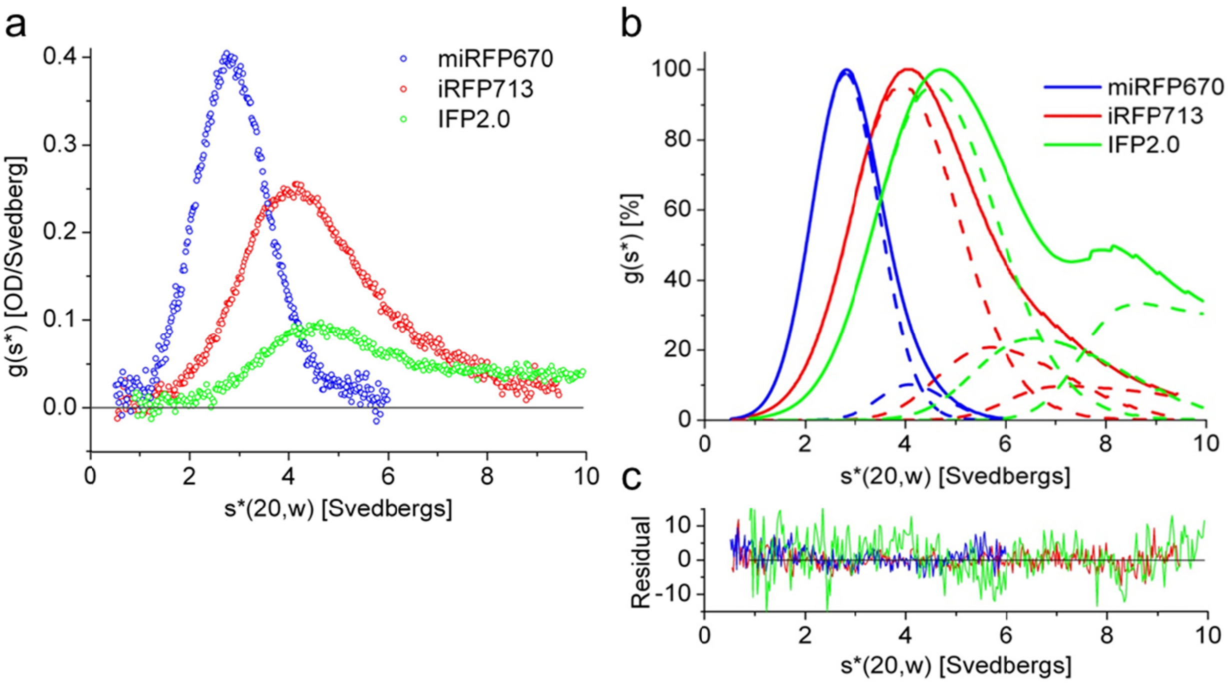

Sedimentation velocity analytical ultracentrifugation study of miRFP670, iRFP713 and IFP2.0 at concentrations of 15 μM run in PBS buffer at 20ºC, analyzed by the time-derivative method. **(a)** Overlay of the sedimentation coefficient distributions for miRFP670 (blue), iRFP713 (red) and IFP2.0 (green). **(b)** Overlay of the normalized best-fit sedimentation coefficient distributions (solid lines) and the individual species that comprise the fits (dashed lines). **(c)** The residuals corresponding to the resolved fits shown in (b). For miRFP670, the peak is centered at a sedimentation coefficient of 2.8 *S* that corresponds to the protein monomer (M_w = 36 ±3 kDa determined from two independent experiments). The monomer-dimer fit model confirms that >90% of miRFP670 is a monomer. For iRFP713 and IFP2.0, the main peaks at ~4.1 *S* correspond to ~60-70% dimers, with the rest of the proteins forming higher oligomers, as can be seen from individual species distributions shown in (b).

Supplementary Figure 5. Effective brightness of miRFP703 and mIFP in mammalian cell lines.

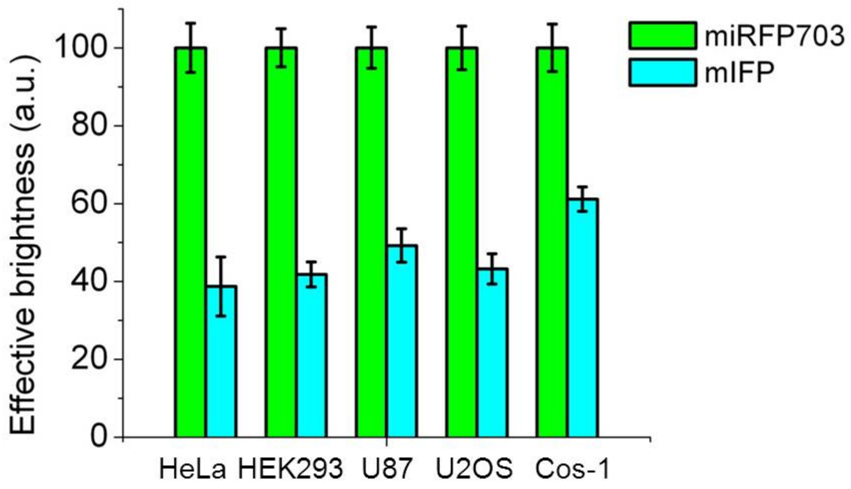

Live HeLa, HEK293, U87, U2OS and Cos-1 cells were transiently transfected with miRFP703 or mIFP. Fluorescence was analyzed by flow cytometry. The NIR fluorescence cell intensity was normalized to that of co-transfected EGFP (to account for differences in transfection efficiency), to excitation efficiency of each NIR FP by 635 nm laser, and to fluorescence signal of each NIR FP in the emission filter. The NIR effective brightness of miRFP703 was assumed to 100% for each cell line. Error bars, s.d. (n=3; transfection experiments).

Supplementary Figure 6. Kinetics of miRFP maturation and chromophore binding.

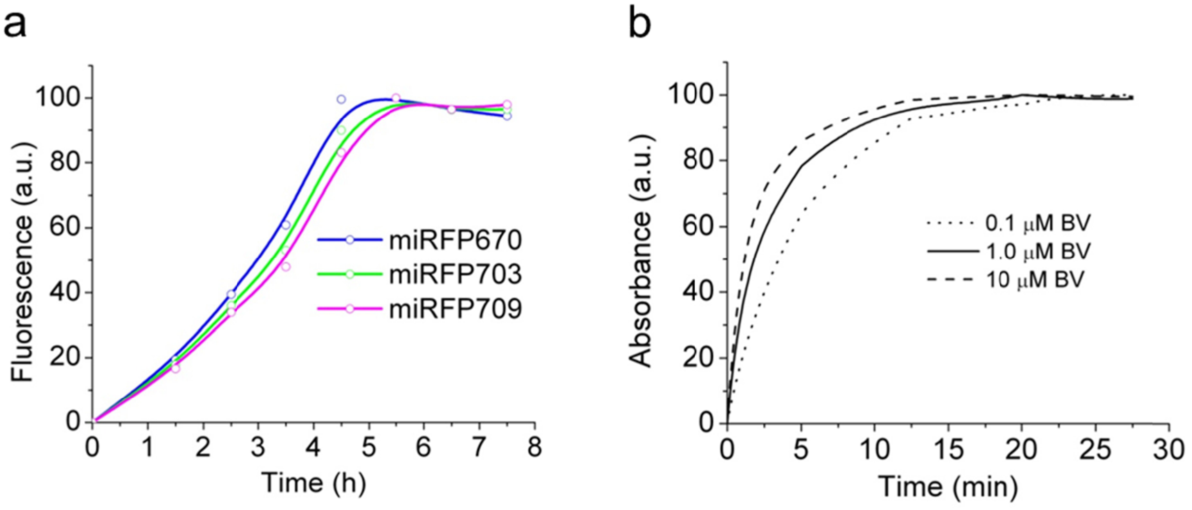

(**a**) Kinetics of miRFP maturation, which is a combination of protein synthesis, folding and chromophore binding, in bacteria grown at 37°C. Time "0" corresponds to the beginning of the 1 h long pulse-chase induction of the protein expression. (**b**) Chromophore binding kinetics measured as assembly of purified miRFP670 apoprotein with BV *in vitro* at 37°C. For this, 15 μM of miRFP670 apoprotein was mixed with different concentrations of free BV in PBS buffer containing 1 mM DTT, and absorbance at the peak was monitored for 30 min.

Supplementary Figure 7. Assay for miRFP cytotoxicity.

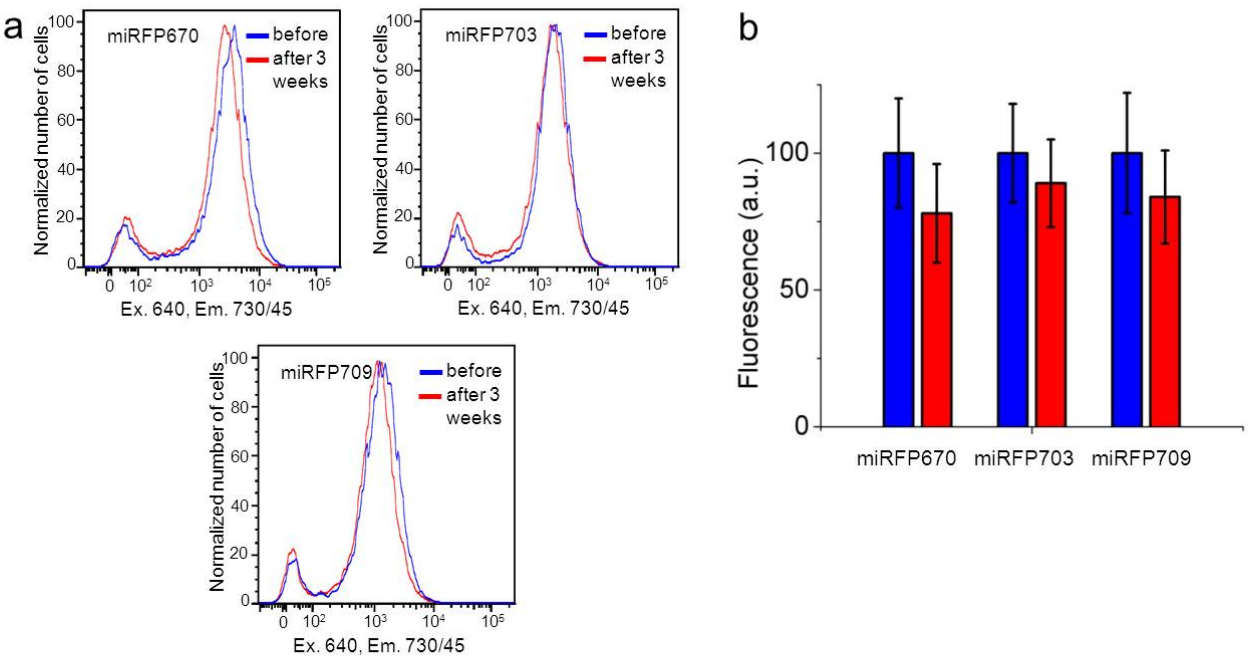

(**a**) Live HEK293 cells stably expressing miRFPs were analyzed by flow cytometry on day 14 (blue) and after 3 weeks (day 35; red) after transfection. Representative cell populations are shown. Number of cells (counts) was normalized to the maximum values for each histogram. Excitation laser and emission filter used for analysis are indicated. (**b**) Mean fluorescence intensities of cells represented in (a). For each FP the values were normalized to the intensity observed on day 14. Error bars, s.d. (n=3).

Supplementary Figure 8. Primary neurons expressing miRFPs.

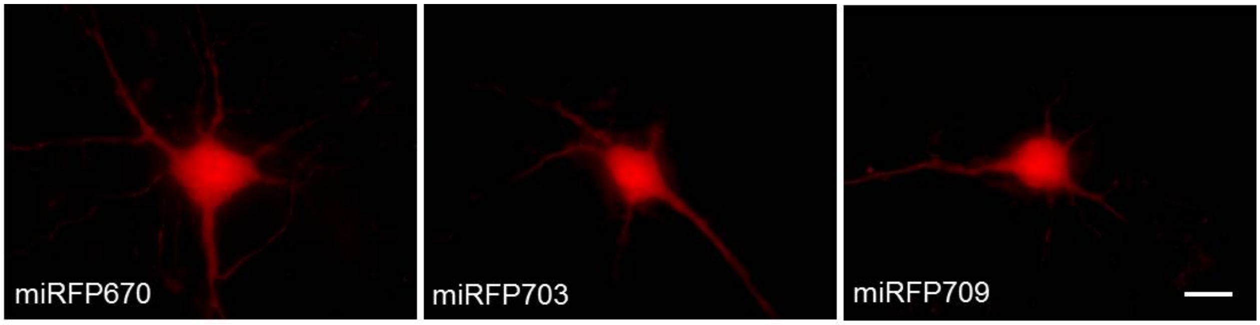

Primary cultures of mouse hippocampal neurons were transfected with miRFP encoding plasmids using Lipofectamine 2000 (Life Technologies/Invitrogen) and imaged 72 h after the transfection. miRFPs in live neurons were imaged using 665/45 nm excitation and 725/50 nm emission filters (red pseudocolor). Scale bar, 10 μm.

Supplementary Figure 9. Two-color imaging of miRFP670 and miRFP709 fusions.

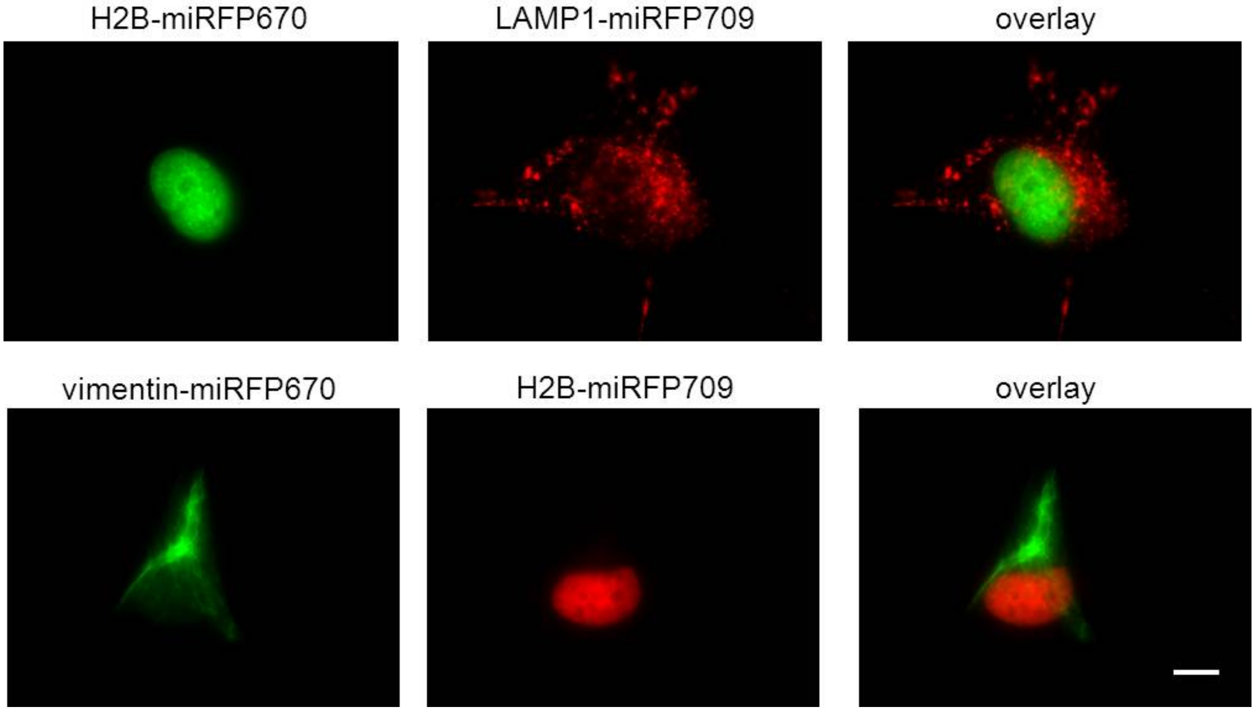

Live HeLa cells co-expressing the indicated miRFP670 and miRFP709 fusions are shown. miRFP670 was imaged using 605/40 nm excitation and 667/30 nm emission filters (green pseudocolor). miRFP709 was imaged using 682/12 nm excitation and 721/42 nm emission filters (red pseudocolor). Scale bar, 10 μm.

Supplementary Figure 10. Imaging of α-tubulin filaments by widefield microscopy and SIM.

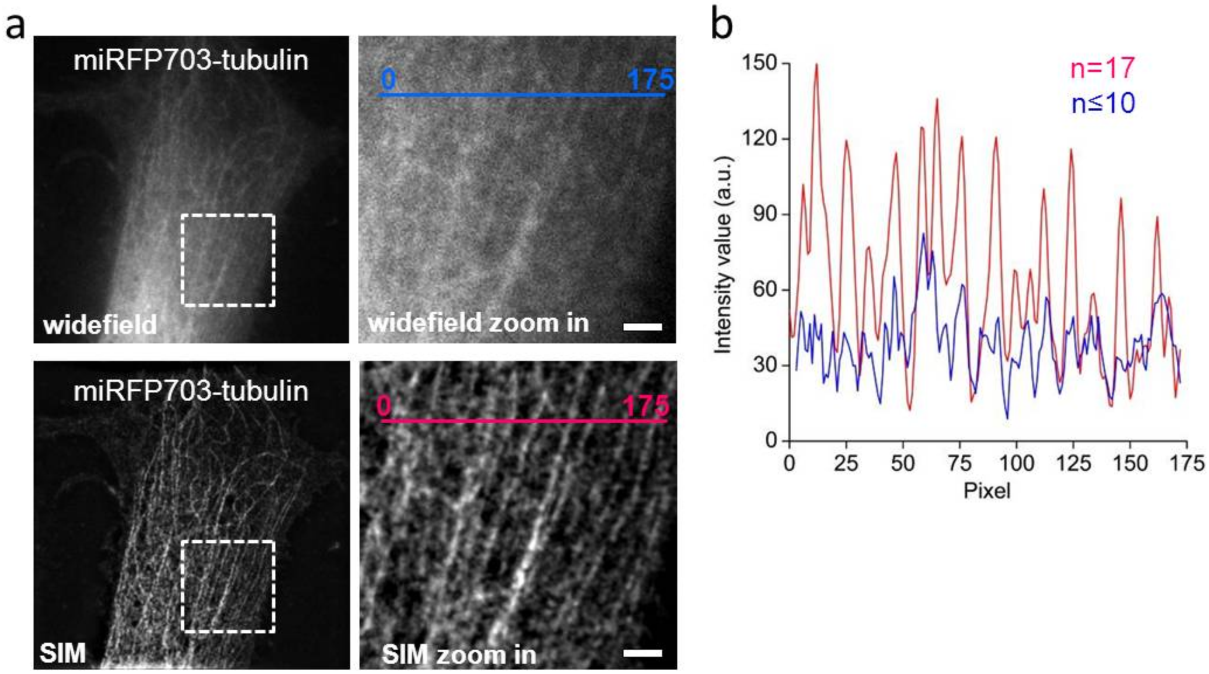

(**a**) Fixed HeLa cells expressing miRFP703-labeled α-tubulin were imaged by widefield microscopy and SIM as shown in Fig. 2b. The magnified regions (on the right) represented by white squares (on the left) are shown. Blue and red lines indicate positions of cross-sections analyzed in (b). Scale bar, 1 μm. (**b**) Intensity profiles of cross-sections shown in the magnified regions in (a). The x-axis numbers correspond to pixels in each intensity profile. The number of filaments spatially resolved in each cross-section is indicated on top right.

Supplementary Figure 11. Kinetics of BiFC contrast increase for miSplit709.

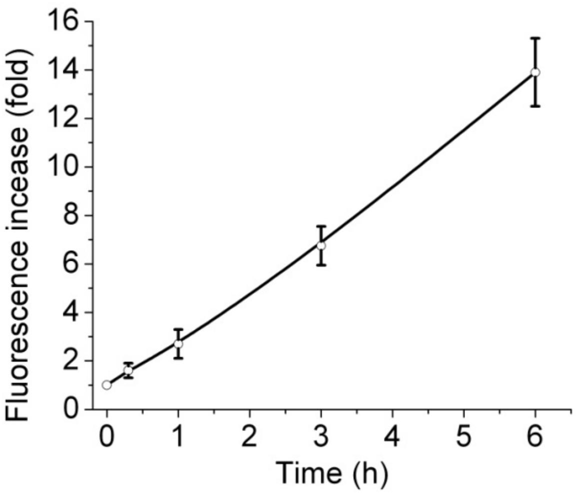

Kinetics of the fluorescence increase in HeLa cells transiently co-transfected with plasmids encoding PAS domain fused with FRB and GAF domain fused with FKBP for miSplit709 after addition of 50 nM rapamycin. The mean fluorescence intensities at each time point were normalized to the fluorescence intensity of cells before addition of rapamycin (time 0 h). The fluorescence intensities were determined using flow cytometry (ex. 640 nm, em. 720/40 nm). Error bars, s.d. (n=3).

Supplementary Figure 12. Brightness and BiFC contrast of miSplits and iSplit.

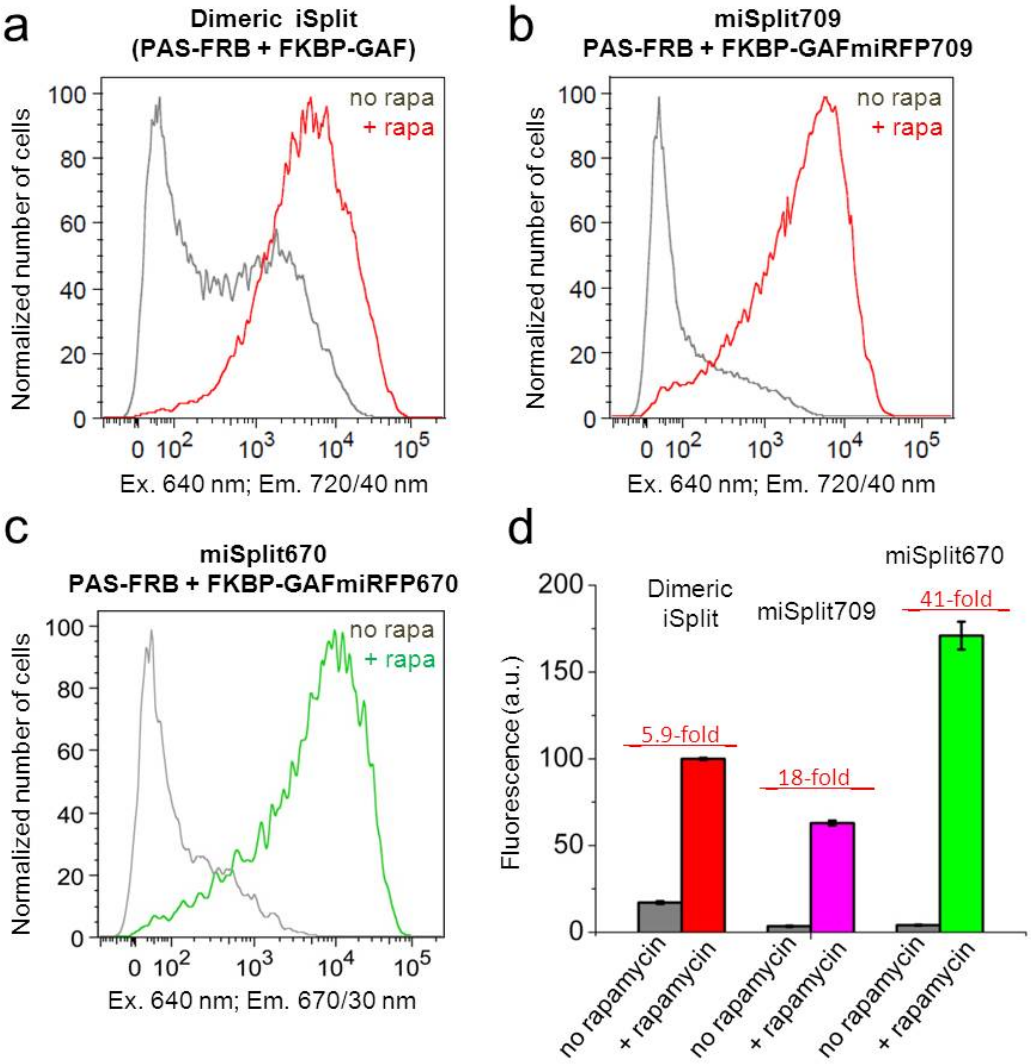

Live HeLa cells transiently transfected with plasmids co-expressing PAS domain fused with FRB and GAF domain fused with FKBP for (**a**) published dimeric iSplit, (**b**) miSplit709 and (**c**) miSplit670 without or with rapamycin (50 nM) were analyzed by flow cytometry. Number of cells (counts) was normalized to the maximum values for each histogram. Excitation laser and emission filters used for analysis are indicated. (**d**) Mean fluorescence intensities of cells represented in (a, b, c). Fluorescence of cells with complemented dimeric iSplit was assumed to 100. BiFC contrast (fold) for each split reporter is indicated. Error bars, s.d. (*n*=3).

Supplementary Figure 13. Detection of mRNA in cells with miSplit670.

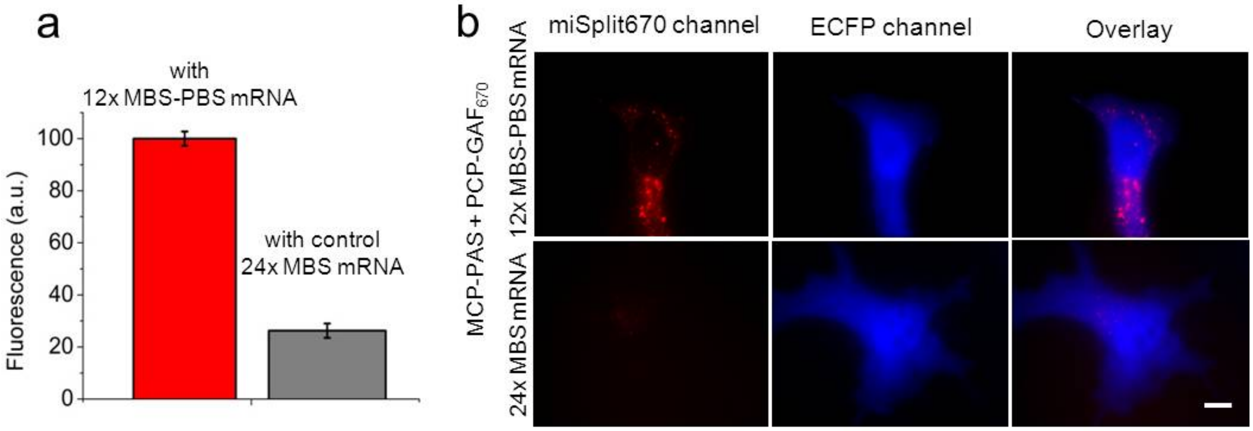

(**a**) Fluorescence of ECFP mRNA labeled with miSplit670 and ECFP mRNA control. Live HeLa cells co-expressed PAS-MCP and PCP-mGAF$_{670}$ together with ECFP mRNA tagged with 12x MBS-PBS binding sites (red). ECFP mRNA tagged with 24x MBS binding sites served as a control (grey). Cell fluorescence was analyzed by flow cytometry. (**b**) Microscopy of live HeLa cells analyzed in (a). Cells were imaged using two filter sets: ex. 605/40 nm and em. 640LP nm to detect miSplit670, and ex. 436/20 nm and em. 455LP nm to detect ECFP. Scale bar, 10 µm.

Supplementary Figure 14. Two-color imaging of IκBα-miRFP670 and H2B-miRFP709.

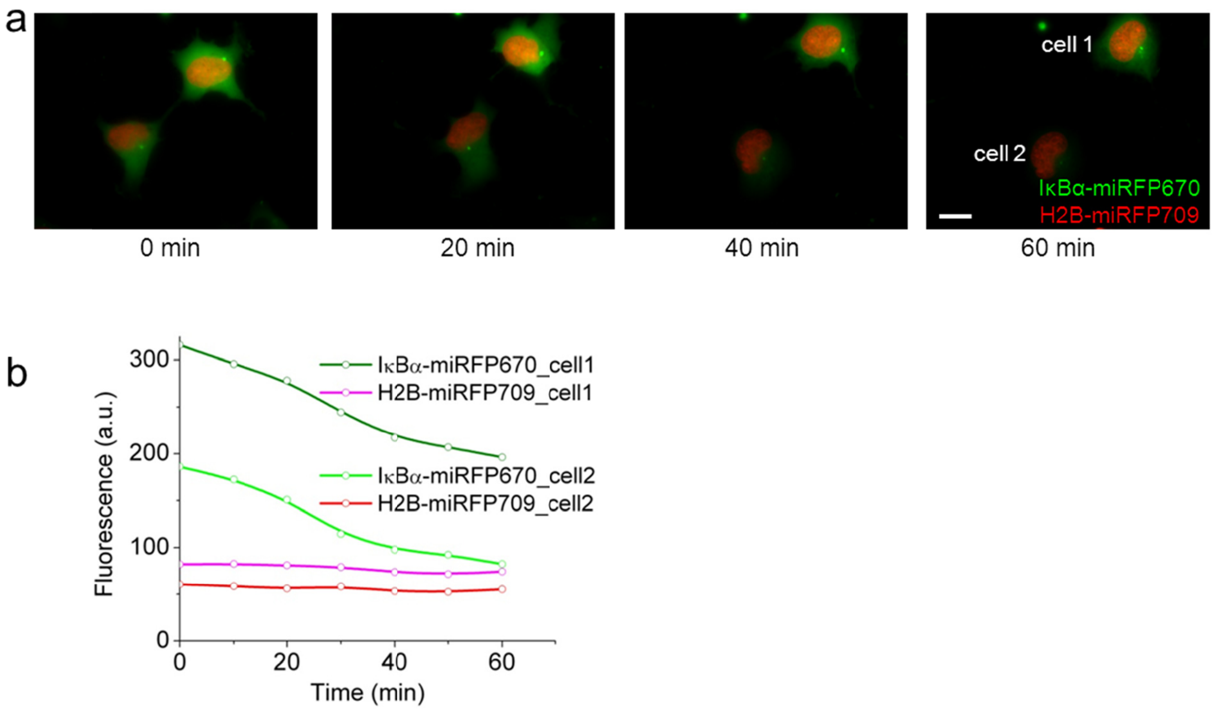

(a) Microscopy time-lapse images of live HEK293 cells transiently co-transfected with plasmids encoding IκBα-miRFP670 (green pseudocolor) and H2B-miRFP709 (red pseudocolor) upon treatment with TNFα. Scale bar, 10 μm. **(b)** Quantification of the fluorescence signals of two cells shown in (a).

Supplementary Figure 15. Analysis of NIR cell cycle reporter using flow cytometry.

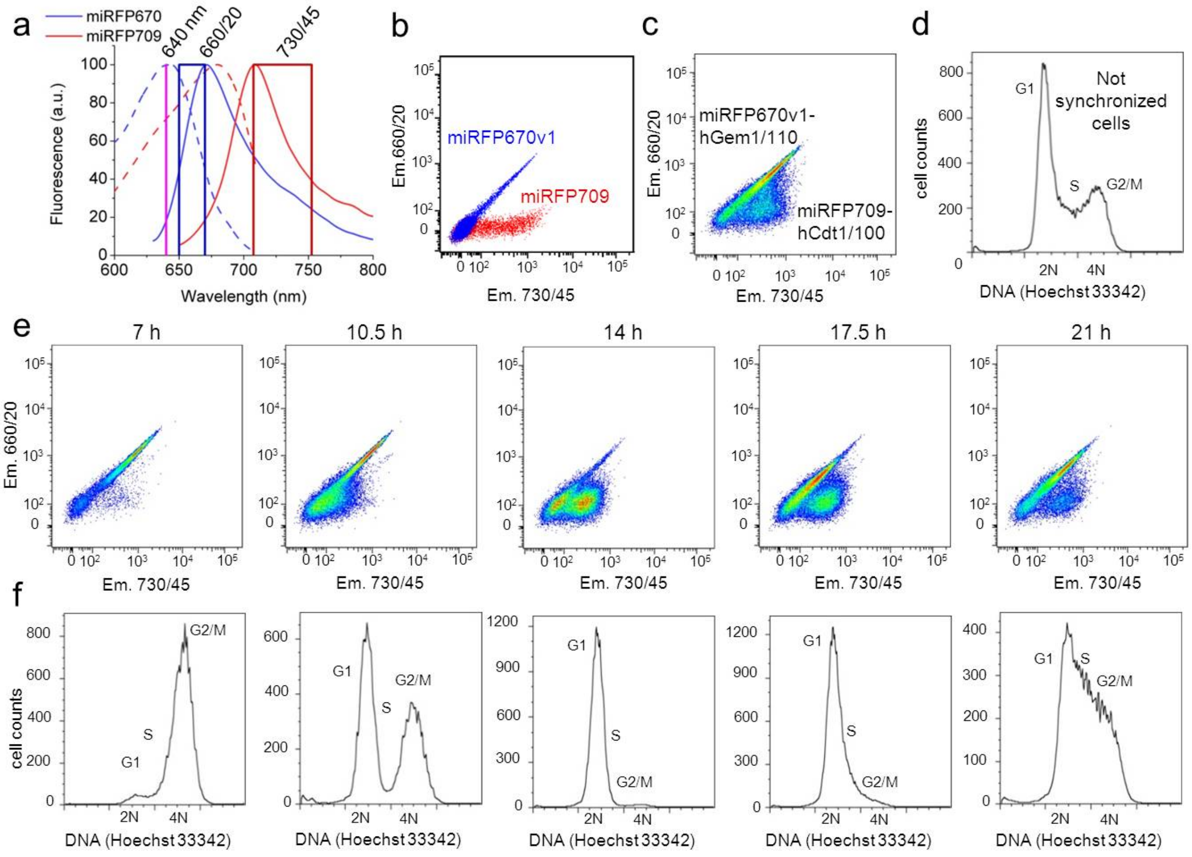

(**a**) Two filter channels used to distinguish between miRFP670 and miRFP709 fluorescence by flow cytometry. The excitation laser and profiles of the two emission filters 660/20 nm and 730/45 nm are overlaid with excitation and emission spectra of miRFPs. (**b**) Separation of cells transiently transfected with miRFP670v1 and miRFP709 using the filter set shown in (a). Live HeLa cells expressing either miRFP670v1 or miRFP709 were analyzed and populations overlaid. (**c**) Flow cytometry analysis of HeLa cells stably co-expressing miRFP670v1-hGem1/100 and miRFP709-hCdt1/110 using the same filter sets as in (b). (**d**) Histograms of Hoechst33342 signal distribution representing the cell cycle progression for the cells shown in (c). (**e**) Flow cytometry histograms corresponding to time points during cell cycle progression. HeLa cells stably co-expressing miRFP670v1-hGem1/100 and miRFP709-hCdt1/110 fusions were released after the synchronization by double thymidine block (similar to cells analyzed by microscopy in Fig. 5b,c). (**f**) Histograms of Hoechst33342 signal distribution representing the cell cycle progression in cells shown in (e).

Supplementary Figure 16. NIR cell cycle reporter in HEK293 and HeLa cells.

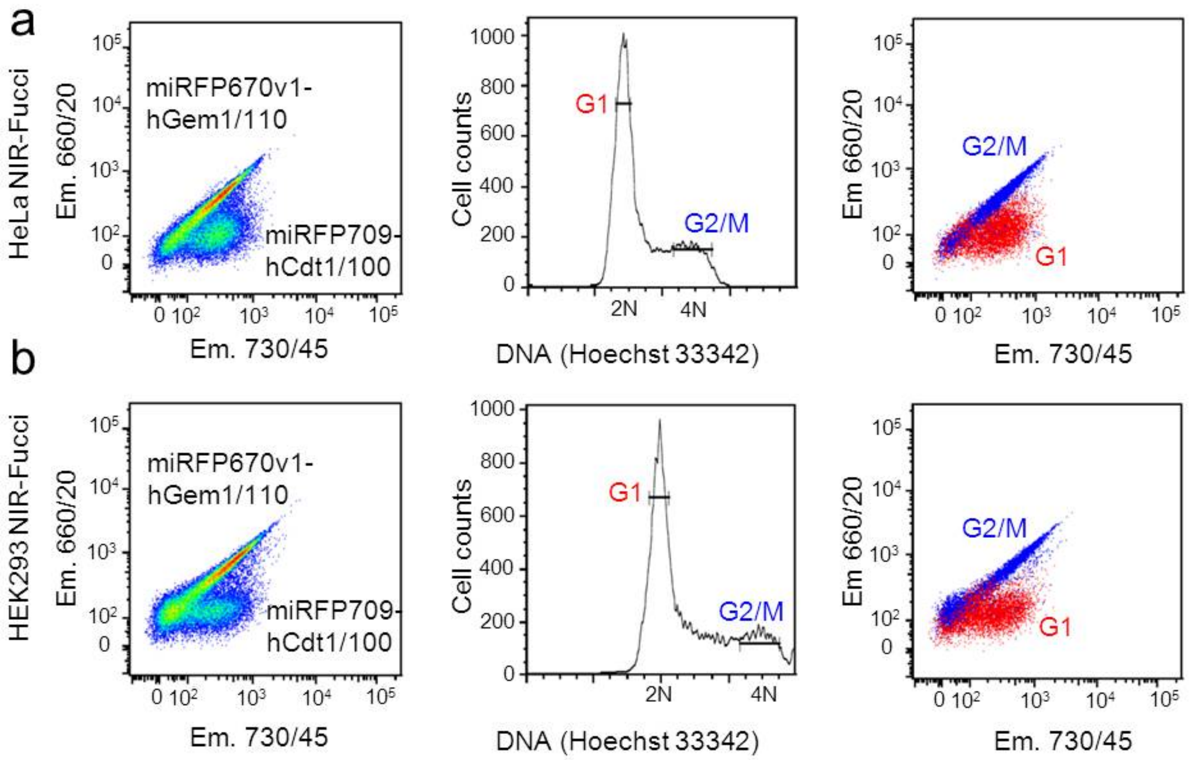

Flow cytometry analysis of (**a**) HeLa cells and (**b**) HEK293 cells stably co-expressing miRFP670v1-hGem1/100 and miRFP709-hCdt1/110. (Left) Flow cytometry histograms shown in two channels allowing to distinguish between miRFP670 and miRFP709 fluorescence. (Middle) Histograms of Hoechst33342 signal distribution representing the cell cycle progression for the cells shown on the left. Two gates corresponding to G1 and G2/M cell cycle phases are created. (Right) An overlay of populations corresponding to two gates shown in the Hoechst histogram (middle). As expected, fluorescence of cells in G2/M phase spectrally corresponds to miRFP670v1, whereas fluorescence of cells in G1 phase spectrally corresponds to miRFP709.

Supplementary Figure 17. Cells and tumors expressing NIR cell cycle reporter in mice.

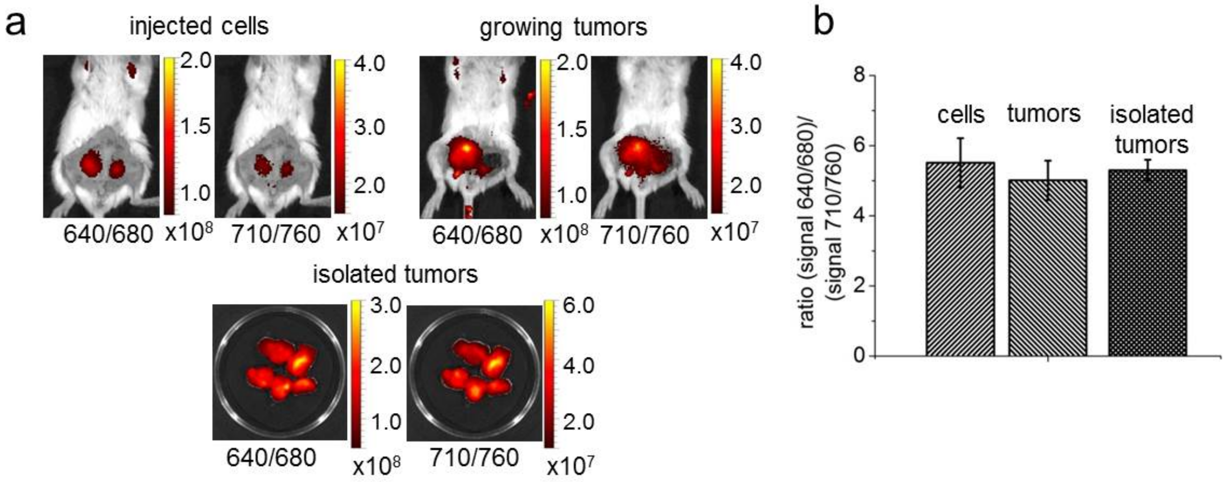

(**a**) Representative images of mice with implanted cells expressing the NIR cell cycle reporter (top left), with the resulted tumors 3 weeks after injection (top right), and isolated tumors (bottom). Two channels, 640/30 nm excitation and 680/20 nm emission for detection of miRFP670v1, and 710/30 nm excitation and 760/20 nm emission for detection of miRFP709, are shown. (**b**) The ratios between fluorescence signals from injected cells in the miRFP670v1 and miRFP709 channels for experiments represented in (a).
